# Design, Synthesis, and Biological Activities of Novel 2-Cyanoacrylate Compounds Containing Substituted Pyrazolyl or 1,2,3-Triazolyl Moiety

**DOI:** 10.3390/molecules28073141

**Published:** 2023-03-31

**Authors:** Yang Wang, Yudie Chen, Ye Qian, Jia Chen, Xianchao Du, Yujun Shi, Baolin Xu, Sheng Hua, Hong Dai

**Affiliations:** College of Chemistry and Chemical Engineering, Nantong University, Nantong 226019, China

**Keywords:** 2-cyanoacrylate, pyrazole, 1,2,3-triazole, synthesis, biological activity

## Abstract

To develop novel 2-cyanoacrylate derivatives with potential bioactivity, a number of 2-cyanoacrylate compounds, including substituted pyrazole or 1,2,3-triazole ring, were designed, prepared, and structurally detected by ^1^H NMR, ^13^C NMR, and elemental analysis. The biological assessment displayed that some designed compounds had significant herbicidal activities against *Brassica juncea*, *Chenopodium serotinum*, *Rumex acetosa*, *Alopecurus aequalis*, *Polypogon fugax,* and *Poa annua* at a dosage of 1500 g/ha. Furthermore, some derivatives still expressed satisfactory herbicidal activities against *Brassica juncea*, *Chenopodium serotinum,* and *Rumex acetosa* when the dosage was lowered to 150 g/ha, especially the inhibitory effects of compounds **9a**, **9d**, **9f**, **9i**, **10a**, **10b**, **10e**, and **10n** against *Brassica juncea* were all over 80%, compounds **9d**, **9f**, **9g**, **9h**, **9i**, **10h**, **10i**, **10m**, **10n**, and **10o** possessed more than 70% inhibition rates against *Chenopodium serotinum*, and compound **9d** indicated 70% herbicidal activity against *Rumex acetosa*. These results provided an important basis for further design and discovery of biologically active 2-cyanoacrylate compounds.

## 1. Introduction

**Figure 1:**
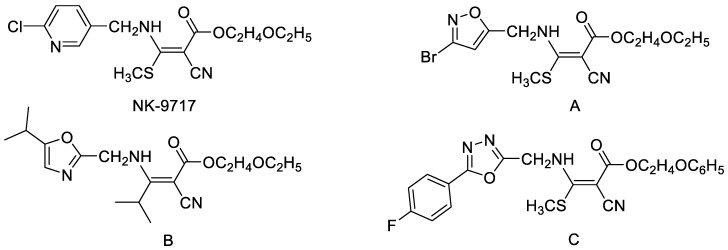
Structures of NK-9717 and compounds **A**–**C**.

In recent years, 2-cyanoacrylatesplay a vital role in pesticidal and medicinal fields. Many 2-cyanoacrylate-based compounds have attracted intense attention for different bioactivities containing insecticidal [1,2], herbicidal [3,4,5], fungicidal [6], plant growth regulatory [7], and antitumor activity [8]. For example, NK-9717 (Figure 1), a potent herbicide owning 2-cyanoacrylate moiety, displayed wonderful herbicidal properties against some weeds such as *Brassica campestris* and *Amaranthus retroflexus* [9]. Recently, Wang et al. reported that isoxazole-containing 2-cyanoacrylate compound A (Figure 1) had significant herbicidal activity [10], Zhao et al. obtained 2-cyanoacrylate compound B (Figure 1) with a substituted oxazole moiety possessing interesting plant growth regulatory effect in addition to excellent herbicidal activity [11]. More recently, Shi and co-workers found that 1,3,4-oxadiazole-containing 2-cyanoacrylate analogue C (Figure 1) exhibited vital antitumor properties against human hepatoma cells [12]. This strongly promoted the research of bioactive molecules containing the 2-cyanoacrylate subunit.

**Figure 2:**
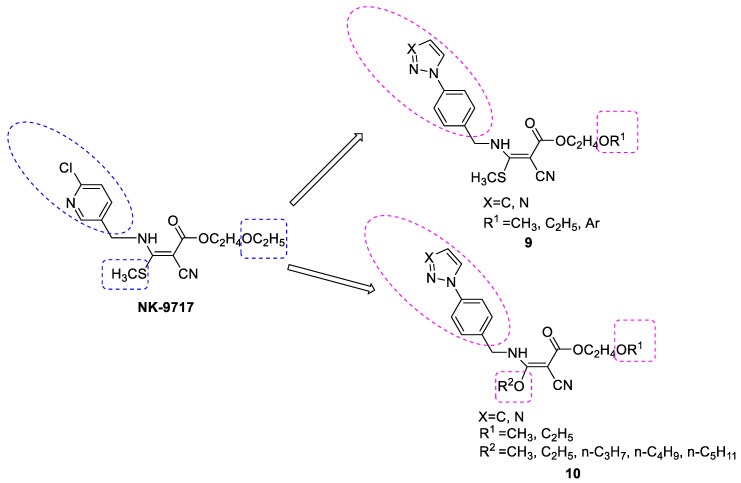
Design strategy of 2-cyanoacrylate derivatives **9** and **10**.

On the other hand, substituted pyrazole and 1,2,3-triazole rings are significant *N*-containing aromatic heterocycles, which act as the main building blocks and intermediates in the synthesis of novel agricultural chemicals and drugs [13,14,15,16]. A number of pyrazole or 1,2,3-triazole derivatives were reported to possess multiple bioactivities and pharmacological properties involving herbicidal [17,18,19], fungicidal [20,21], insecticidal [22,23], anti-proliferative [24,25], antibacterial [26,27], and anticancer effects [28,29,30]. In light of the above reports, we hypothesized that the introduction of privileged pyrazole or 1,2,3-triazole pharmacophore to the parent 2-cyanoacrylate skeleton might generate some new bioactive compounds (Figure 2). Herein, we report the design and preparation of new 2-cyanoacrylate derivatives carrying pyrazolyl or 1,2,3-triazolyl moiety. Moreover, the newly synthesized 2-cyanoacrylate compounds were investigated for their herbicidal activities.

## 2. Results and Discussion

### 2.1. Chemistry

**Scheme 1:**
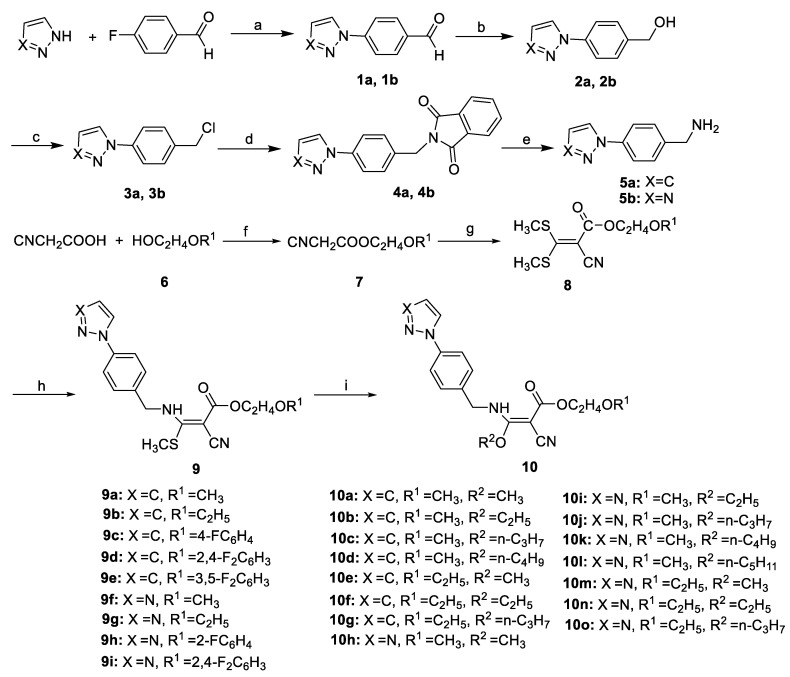
Synthesis of the designed compounds **9a**–**9i** and **10a**–**10o**. *Reagents and conditions:* (a) potassium carbonate, DMF, 120 °C, 8 h; (b) NaBH_4_, C_2_H_5_OH, 0 °C, 5 h; (c) thionyl chloride, CH_2_Cl_2_, 0 °C, 30 min, r.t., 6 h; (d) potassium phthalimide, DMF, 50 °C, 9 h; (e) hydrazine hydrate, C_2_H_5_OH, reflux, 3 h; (f) sodium bisulfate monohydrate or condensed sulfuric acid, toluene, reflux, 8–10 h; (g) potassium hydroxide, carbon disulfide, acetonitrile, 0 °C, 4 h, and then dimethyl sulfate, r.t., 10–12 h; (h) compounds **5**, C_2_H_5_OH, reflux, 4–6 h; (i) R^2^ONa, R^2^OH, 0 °C, 4–5 h.

In this paper, in order to search for novel 2-cyanoacrylates with wonderful bioactivities, NK-9717 was chosen as the pesticide precursor, and the active pyrazolyl and 1,2,3-triazolyl units were introduced to replace the pyridyl ring to design and prepare new 2-cyanoacrylate compounds **9** and **10** (Figure 2). The synthesis process of the title compounds **9** and **10** was depicted in Scheme 1. The condensation of 4-fluorobenzaldehyde with 1*H*-pyrazole or 1*H*-1,2,3-triazole under alkaline conditions offered compounds **1**, which were conveniently reduced to compounds **2** by treatment with NaBH_4_. Furthermore, intermediates **2** reacted with thionyl chloride to produce compounds **3** successfully. Intermediates **3** were easily converted into compounds **4** by the reaction with potassium phthalimide, which were further transformed into compounds **5** by the treatment with hydrazine hydrate. The key intermediates **8** were easily synthesized in two steps from compounds **6**. Cyanoacetic acid was treated with 2-alkoxy or 2-aryloxy alcohols **6** to generate intermediates **7**, which were reacted with carbon disulfide and dimethyl sulfate to obtain compounds **8** directly. Subsequently, the significant intermediates **5** were mixed with 2-cyanoacrylates **8** in ethanol to obtain the target compounds **9a**–**9i** in satisfactory yields. At the same time, compounds **9** reacted with sodium alcoholate to form the aimed compounds **10a**–**10o** successfully. Finally, the structures of compounds **9a**–**9i** and **10a**–**10o** were determined through ^1^H NMR, ^13^C NMR, and elemental analysis. Compounds **9a** and **10b** were selected as the typical examples to illustrate. In ^1^H NMR of **9a**, the proton signal peak of the NH group was displayed at *δ* 10.36, a doublet signal at *δ* 4.80 was due to two protons of methylene group that attached to a nitrogen atom, and three protons of methylthio unit were detected as a singlet signal at *δ* 2.68. In ^13^C NMR of **9a**, the carbon atom of the C=O group was noticed at *δ* 168.26, the carbon atom of the methylene group that attached to nitrogen was recognized at *δ* 48.95, and the carbon atom of methylthio moiety was shown at *δ* 18.33. In ^1^H NMR of **10b**, one proton of NH moiety was detected at *δ* 9.78, a quartet signal at *δ* 4.61 belongs to two protons of methyleneoxy moiety adjacent to C=C group, and two protons of the methylene unit linked to nitrogen atom was exhibited as a singlet signal at *δ* 4.53. In ^13^C NMR of **10b**, the C=O group was shown at *δ* 169.63, the carbon atom of methyleneoxy unit adjacent to the C=C skeleton was noticed at *δ* 59.86, and the carbon atom of methylene moiety attached to nitrogen was seen at *δ* 44.53. 

### 2.2. Biological Activities

The herbicidal activities of 2-cyanoacrylates **9a**–**9i** and **10a**–**10o** were tested towards *Brassica juncea*, *Chenopodium serotinum*, *Rumex acetosa*, *Alopecurus aequalis*, *Polypogon fugax,* and *Poa annua*. Related data were summarized in Table 1; most target compounds indicated satisfactory herbicidal activity against *Brassica juncea* at 1500 g/ha, the inhibition effects of **9a**, **9d**, **9f**, **9g**, **9h**, **9i**, **10a**, **10b**, **10e**, **10h**, **10l**, **10m**, **10n**, and **10o** against *Brassica juncea* were over 90%, respectively, which were equal to that of NK-9717 (100%). At 1500 g/ha, most of the target compounds also had wonderful herbicidal activity against *Chenopodium serotinum*; for instance, compounds **9a**, **9d**, **9f**, **9g**, **9h**, **9i**, **10a**, **10e**, **10f**, **10h**, **10i**, **10m**, **10n**, and **10o** owned > 90% inhibition effects, which were near to that of the control NK-9717 (100%). At 1500 g/ha, some compounds possessed moderate to good inhibition rates against *Rumex acetosa*, especially compounds **9a**, **9d**, **10e**, **10f**, and **10n,** and all had 100% inhibition effects against *Rumex acetosa*, which were close to that of NK-9717. At the same time, some title compounds were active against *Alopecurus aequalis* at a dosage of 1500 g/ha, and the inhibition rates of compounds **9a**, **9d**, **9g**, **9h**, **9i**, **10a**, **10e**, **10h**, **10i**, **10m**, **10n**, and **10o** against *Alopecurus aequalis* were 45%, 50%, 70%, 50%, 70%, 80%, 50%, 85%, 70%, 70%, 100%, and 90%, which surpassed that of NK-9717 (30%). Additionally, some 2-cyanoacrylates such as **9a**, **9d**, **9g**, **9h**, **9i**, **10a**, **10e**, **10h**, **10i**, **10m**, and **10n** showed potent herbicidal activity against *Polypogon fugax* at 1500 g/ha with inhibitory values of 40%, 40%, 50%, 40%, 70%, 45%, 80%, 70%, 60%, and 100%, respectively, which were superior to that of the control NK-9717 (20%). Interestingly, some of these derivatives still displayed certain inhibition effects against *Poa annu* at 1500 g/ha. Among them, compounds **10h**, **10m**, and **10n** owned 50%, 40%, and 70% inhibitory values, respectively, which were much better than that of the control NK-9717 (10%). From the data listed in Table 1, we found that when X is a C atom, among the 3-methylthio compounds **9a**, **9b**, **9c**, **9d**, and **9e**, the inhibitory values of **9a** (R^1^ = methyl) against *Brassica juncea*, *Chenopodium serotinum*, *Rumex acetosa*, *Alopecurus aequalis*, *Polypogon fugax,* and *Poa annua* were much better than those of **9b** (R^1^ = ethyl), and the inhibition effects of **9d** (R^1^ = 2,4-F_2_C_6_H_3_) against *Brassica juncea*, *Chenopodium serotinum*, *Rumex acetosa*, *Alopecurus aequalis*, *Polypogon fugax,* and *Poa annua* were superior to those of **9c** (R^1^ = 4-FC_6_H_4_) and **9e** (R^1^ = 3,5-F_2_C_6_H_3_). Among 3-methylthio derivatives, when R^1^ is ethyl, compound **9g** (X = N) demonstrated higher inhibitory values against *Brassica juncea*, *Chenopodium serotinum*, *Rumex acetosa*, *Alopecurus aequalis*, *Polypogon fugax,* and *Poa annua* than compound **9b** (X = C). Among 3-alkoxy derivatives **10a**–**10g** (X = C), when R^1^ is methyl, compounds **10a** (R^2^ = methyl) and **10b** (R^2^ = ethyl) exhibited better inhibition rates against *Brassica juncea*, *Chenopodium serotinum*, *Rumex acetosa*, *Alopecurus aequalis*, *Polypogon fugax,* and *Poa annua* than compounds **10c** (R^2^ = n-propyl) and **10d** (R^2^ = n-butyl), and when R^1^ is ethyl, compounds **10e** (R^2^ = methyl) and **10f** (R^2^ = ethyl) possessed higher inhibition rates against *Brassica juncea*, *Chenopodium serotinum,* and *Rumex acetosa* than compounds **10g** (R^2^ = n-propyl). Among 3-alkoxy derivatives **10h**–**10o** (X = N), when R^1^ is methyl, the herbicidal activities of compounds **10h** (R^2^ = methyl) and **10i** (R^2^ = ethyl) against *Chenopodium serotinum*, *Alopecurus aequalis*, *Polypogon fugax,* and *Poa annua* were superior to those of compounds **10j** (R^1^ = n-propyl), **10k** (R^1^ = n-butyl), and **10l** (R^1^ = n-pentyl), and when R^1^ is ethyl, compound **10n** (R^2^ = ethyl) was more active against *Rumex acetosa*, *Polypogon fugax* and *Poa annua* than compounds **10m** (R^2^ = methyl) and **10o** (R^2^ = n-propyl). Herbicidal activities of some designed compounds at 150 g/ha were presented in Table 2. Among the 3-methylthio compounds **9a**, **9d**, **9f**–**9i**, all of them exhibited important inhibition rates against *Brassica juncea* at 150 g/ha, especially compounds **9a**, **9d**, **9f**, and **9i** had over 80% inhibition effects against *Brassica juncea*, which were close to that of the control NK-9717. Compounds **9d**, **9f**, **9g**, and **9i** showed 90%, 80%, 80%, and 80% inhibitory values against *Chenopodium serotinum*, which were better than that of NK-9717 (75%). Compound **9d** demonstrated a 70% inhibitory value against *Rumex acetosa*, which was higher than that of NK-9717 (40%). Among the 3-alkoxy compounds, **10a**, **10b**, **10e**, **10h**, **10m**, and **10n** displayed wonderful herbicidal activity against *Brassica juncea* with inhibitory rates of 80%, 80%, 80%, 75%, 75%, and 80%, which were close to NK-9717 (80%). Compounds **10h**, **10i**, **10m**, **10n**, and **10o** indicated 70%, 75%, 85%, 80%, and 70% herbicidal properties against *Chenopodium serotinum*, which were near to that of NK-9717 (75%). Additionally, Compounds **10n** and **10o** both expressed 40% inhibition effects against *Rumex acetosa*, which were equal to that of NK-9717 (40%). From the bioactive data depicted in Table 1 and Table 2, we could find that the bioactivity spectra of 2-cyanoacrylates were greatly improved by importing the significant pyrazole and 1,2,3-triazole moieties to the lead compound NK-9717, especially some of these designed compounds displayed better herbicidal activities against *Alopecurus aequalis*, *Polypogon fugax,* and *Poa annua* than those of NK-9717, which implied that the introduction of vital pyrazole and 1,2,3-triazole frameworks was beneficial to broaden the herbicidal properties of 2-cyanoacrylate derivatives. Compounds **9g** (X = N, R^1^ = ethyl), **10i** (X = N, R^1^ = methyl, R^2^ = ethyl), and **10n** (X = N, R^1^ = ethyl, R^2^ = ethyl) possessed extensive herbicidal properties against all the tested weeds, which could be selected as the bioactive compounds for further structural modification and bioactivity study. 

## 3. Materials and Methods

### 3.1. Chemistry

Chemical agents were commercially available and treated through standard methods. ^1^H NMR, and ^13^C NMR spectra were detected by Bruker AV400 spectrometer (400 MHz, ^1^H; 100 MHz, ^13^C, Bruker, Billerica, MA, USA) in the solvent of CDCl_3_ or DMSO-*d*_6_. Melting points (M.p.) were performed using an X-4 apparatus. Elemental analysis was provided by a Yanaco CHN Corder MT-3 elemental analyser (Yanaco, Kyoto, Japan). Intermediates **1**–**3** were synthesized with reported procedures [31]. Intermediates **6**–**8** were prepared according to the literature method [32]. 

#### 3.1.1. General Approach to Preparation of **4**

Compounds **3** (5 mmol) were dissolved in 30 mL of DMF, and potassium phthalimide (6 mmol) was directly added thereto at room temperature. Next, the mixture was maintained at 50 °C for another 9 h. After cooling, the solution was dumped into 50 mL of ice water. Finally, the precipitates were filtered to get intermediates **4**. Compound **4a**: colorless solid (76% yield), m.p. 161–163 °C; ^1^H NMR (CDCl_3_) *δ* 7.88 (d, *J* = 2.40 Hz, 1H), 7.84–7.86 (m, 2H), 7.63–7.72 (m, 5H), 7.53 (d, *J* = 8.42 Hz, 2H), 6.45 (t, *J* = 2.40 Hz, 1H), 4.87 (s, 2H); ^13^C NMR (CDCl_3_) *δ* 168.04, 141.18, 139.73, 134.55, 134.11, 132.05, 129.89, 126.74, 123.44, 119.33, 107.72, 41.06. Anal. Calcd. for C_18_H_13_N_3_O_2_: C 71.28; H 4.32; N 13.85. Found: C 71.39; H 4.19; N 13.69. Compound **4b**: colorless solid (70% yield), m.p. 189–190 °C; ^1^H NMR (CDCl_3_) *δ* 8.03 (d, *J* = 8.40 Hz, 2H), 7.85–7.87 (m, 2H), 7.79 (s, 2H), 7.71–7.73 (m, 2H), 7.56 (d, *J* = 8.42 Hz, 2H), 4.89 (s, 2H); ^13^C NMR (CDCl_3_) *δ* 168.03, 139.36, 135.70, 135.61, 134.14, 132.03, 129.71, 123.47, 119.15, 41.07. Anal. Calcd. for C_17_H_12_N_4_O_2_: C 67.10; H 3.97; N 18.41. Found: C 67.25; H 3.85; N 18.53. ^1^H NMR and ^13^C NMR spectra are provided in Supplementary Materials.

#### 3.1.2. General Approach to Preparation of **5**

Compounds **4** (3 mmol) were dissolved in 60 mL of anhydrous ethanol, and hydrazine hydrate (80%, 6 mmol) was slowly added thereto. Then, the above solution was kept at reflux for 3 h. After filtration, the mother liquor was condensed to afford intermediates **5**. Compound **5a**: colorless solid (85% yield), m.p. 129–130 °C; ^1^H NMR (DMSO-*d*_6_) *δ* 8.47 (s, 1H), 7.44–7.78 (m, 5H), 6.53 (s, 1H), 3.76 (s, 2H), 3.11 (s, 2H); ^13^C NMR (DMSO-*d*_6_) *δ* 142.26, 141.16, 138.64, 128.59, 128.01, 118.61, 108.12, 45.35. Anal. Calcd. for C_10_H_11_N_3_: C 69.34; H 6.40; N 24.26. Found: C 69.21; H 6.32; N 24.41. Compound **5b**: colorless solid (83% yield), m.p. 169–171 °C; ^1^H NMR (DMSO-*d*_6_) *δ* 8.82 (s, 1H), 7.55–7.98 (m, 5H), 3.81 (s, 2H), 3.09 (s, 2H); ^13^C NMR (DMSO-*d*_6_) *δ* 144.95, 135.46, 134.83, 128.81, 123.56, 120.34, 45.36. Anal. Calcd. for C_9_H_10_N_4_: C 62.05; H 5.79; N 32.16. Found: C 62.19; H 5.68; N 32.08. ^1^H NMR and ^13^C NMR spectra are given in Supplementary Materials. 

#### 3.1.3. General Approach to Preparation of Title Compounds **9a**–**9i**

Compounds **8** (2 mmol) were dissolved in anhydrous ethanol (35 mL), and intermediate **5** (2 mmol) was put in the above solution. Next, the mixture was maintained at reflux for 4–6 h. When the reaction was finished, the resulting solution was condensed to form crude products, which were further refined through column chromatography and washed by ethyl acetate/petroleum ether (1:4, *v*/*v*) to obtain compounds **9a**–**9i**, and the spectral data of compounds **9a**–**9i** are depicted below. ^1^H NMR and ^13^C NMR spectra are listed in Supplementary Materials. 

Data for **9a**: yellow solid (64% yield), m.p. 71–73 °C; ^1^H NMR (CDCl_3_) *δ* 10.36 (s, 1H), 7.93 (d, *J* = 2.40 Hz, 1H), 7.70–7.73 (m, 3H), 7.34 (d, *J* = 8.42 Hz, 2H), 6.48 (t, *J* = 2.40 Hz, 1H), 4.80 (d, *J* = 5.60 Hz, 2H), 4.31 (t, *J* = 4.80 Hz, 2H), 3.66 (t, *J* = 4.80 Hz, 2H), 3.41 (s, 3H), 2.68 (s, 3H); ^13^C NMR (CDCl_3_) *δ* 172.76, 168.26, 141.32, 139.97, 134.52, 128.36, 126.73, 119.66, 118.06, 107.91, 75.32, 70.30, 63.92, 59.27, 48.95, 18.33. Anal. Calcd. for C_18_H_20_N_4_O_3_S: C 58.05; H 5.41; N 15.04.Found: C 58.19; H 5.29; N 15.17.

Data for **9b**: yellow solid (65% yield), m.p. 79–81 °C; ^1^H NMR (CDCl_3_) *δ* 10.37 (s, 1H), 7.93 (d, *J* = 2.40 Hz, 1H), 7.70–7.74 (m, 3H), 7.33 (d, *J* = 8.42 Hz, 2H), 6.48 (t, *J* = 2.40 Hz, 1H), 4.81 (d, *J* = 5.60 Hz, 2H), 4.30 (t, *J* = 5.00 Hz, 2H), 3.70 (t, *J* = 5.00 Hz, 2H), 3.58 (q, *J* = 6.80 Hz, 2H), 2.67 (s, 3H), 1.21 (t, *J* = 6.80 Hz, 3H); ^13^C NMR (CDCl_3_) *δ* 172.71, 168.27, 141.32, 139.95, 134.56, 128.34, 126.73, 119.66, 118.04, 107.91, 75.40, 68.09, 66.87, 64.15, 48.93, 18.33, 15.17. Anal. Calcd. for C_19_H_22_N_4_O_3_S: C 59.05; H 5.74; N 14.50. Found: C 58.90; H 5.86; N 14.63.

Data for **9c**: colorless solid (62% yield), m.p. 103–105 °C; ^1^H NMR (CDCl_3_) *δ* 10.34 (s, 1H), 7.93 (d, *J* = 1.60 Hz, 1H), 7.71–7.74 (m,3H), 7.34 (d, *J* = 8.02 Hz, 2H), 6.85–6.98 (m, 4H), 6.49 (s, 1H), 4.81 (d, *J* = 5.60 Hz, 2H), 4.48 (t, *J* = 4.80 Hz, 2H), 4.19 (t, *J* = 5.00 Hz, 2H), 2.68 (s, 3H); ^13^C NMR (CDCl_3_) *δ* 173.01, 168.14, 158.74, 156.36, 154.66, 141.32, 139.95, 134.46, 128.39, 126.77, 119.71, 117.95, 116.10, 116.02, 115.75, 107.96, 75.07, 66.68, 63.00, 49.00, 18.34. Anal. Calcd. for C_23_H_21_FN_4_O_3_S: C 61.05; H 4.68; N 12.38. Found: C 61.18; H 4.53; N 12.24.

Data for **9d**: yellow solid (61% yield), m.p. 93–95 °C; ^1^H NMR (CDCl_3_) *δ* 10.34 (s, 1H), 7.93 (d, *J* = 2.40 Hz, 1H), 7.71–7.74 (m, 3H), 7.34 (d, *J* = 8.82 Hz, 2H), 7.00–7.06 (m, 1H), 6.79–6.87 (m, 2H), 6.49 (t, *J* = 2.00 Hz, 1H), 4.81 (d, *J* = 6.00 Hz, 2H), 4.50 (t, *J* = 4.80 Hz, 2H), 4.28 (t, *J* = 5.20 Hz, 2H), 2.69 (s, 3H); ^13^C NMR (CDCl_3_) *δ* 173.05, 168.06, 158.37, 155.96, 154.33, 151.85, 143.11, 141.32, 139.96, 134.45, 128.37, 126.76, 119.69, 117.93, 117.67, 110.78, 107.94, 104.96, 75.00, 68.72, 62.95, 49.00, 18.34. Anal. Calcd. for C_23_H_20_F_2_N_4_O_3_S: C 58.72; H 4.28; N 11.91. Found: C 58.57; H 4.43; N 11.99.

Data for **9e**: yellow solid, (61% yield), m.p. 91–93 °C; ^1^H NMR (CDCl_3_) *δ* 10.34 (s, 1H), 7.93 (d, *J* = 2.00 Hz, 1H), 7.73 (d, *J* = 8.42 Hz, 3H), 7.35 (d, *J* = 8.38 Hz, 2H), 6.42–6.49 (m, 4H), 4.82 (d, *J* = 5.60 Hz, 2H), 4.49 (t, *J* = 5.20 Hz, 2H), 4.19 (t, *J* = 5.20 Hz, 2H), 2.69 (s, 3H); ^13^C NMR (CDCl_3_) *δ* 173.17, 168.08, 164.94, 162.49, 160.35, 141.34, 140.00, 134.38, 128.40, 126.77, 119.72, 117.86, 107.96, 98.73, 98.45, 96.81, 74.87, 66.36, 62.45, 49.03, 18.34. Anal. Calcd. for C_23_H_20_F_2_N_4_O_3_S: C 58.72; H 4.28; N 11.91. Found: C 58.86; H 4.16; N 11.78.

Data for **9f**: colorless solid (55% yield), m.p. 100–102 °C; ^1^H NMR (CDCl_3_) *δ* 10.40 (s, 1H), 8.10 (d, *J* = 8.40 Hz, 2H), 7.83 (s, 2H), 7.37 (d, *J* = 8.42 Hz, 2H), 4.83 (d, *J* = 6.00 Hz, 2H), 4.32 (t, *J* = 5.20 Hz, 2H), 3.66 (t, *J* = 5.20 Hz, 2H), 3.41(s, 3H), 2.68 (s, 3H); ^13^C NMR (CDCl_3_) *δ* 172.83, 168.25, 139.54, 135.75, 135.71, 128.17, 119.48, 118.08, 75.30, 70.28, 63.92, 59.29, 48.91, 18.34. Anal. Calcd. for C_17_H_19_N_5_O_3_S: C 54.68; H 5.13; N 18.75. Found: C 54.75; H 5.22; N 18.63.

Data for **9g**: colorless solid (48% yield), m.p. 105–107 °C; ^1^H NMR (CDCl_3_) *δ* 10.40 (s, 1H), 8.11 (d, *J* = 8.40 Hz, 2H), 7.83 (s, 2H), 7.37 (d, *J* = 8.42 Hz, 2H), 4.83 (d, *J* = 5.60 Hz, 2H), 4.30 (t, *J* = 4.80 Hz, 2H), 3.71 (t, *J* = 5.20 Hz, 2H), 3.57 (q, *J* = 6.80 Hz, 2H), 2.67 (s, 3H), 1.22 (t, *J* = 6.80 Hz, 3H); ^13^C NMR (CDCl_3_) *δ* 172.79, 168.25, 139.53, 135.75, 128.14, 119.48, 118.07, 75.40, 68.07, 66.87, 64.15, 48.89, 18.34, 15.17. Anal. Calcd. for C_18_H_21_N_5_O_3_S: C 55.80; H 5.46; N 18.08. Found: C 55.67; H 5.57; N 18.20.

Data for **9h**: colorless solid (60% yield), m.p. 122–124 °C; ^1^H NMR (CDCl_3_) *δ* 10.38 (s, 1H), 8.11 (d, *J* = 8.82 Hz, 2H), 7.83 (s, 2H), 7.38 (d, *J* = 8.82 Hz, 2H), 6.91–7.09 (m, 4H), 4.84 (d, *J* = 6.00 Hz, 2H), 4.54 (t, *J* = 4.80 Hz, 2H), 4.31 (t, *J* = 4.80 Hz, 2H), 2.68 (s, 3H); ^13^C NMR (CDCl_3_) *δ* 173.08, 168.05, 154.16, 151.72, 146.52, 139.55, 135.75, 135.66, 128.15, 124.41, 121.97, 121.90, 119.49, 117.95, 116.44, 116.10, 75.08, 67.50, 62.86, 48.94, 18.35. Anal. Calcd. for C_22_H_20_FN_5_O_3_S: C 58.27; H 4.45; N 15.44. Found: C 58.38; H 4.32; N 15.53.

Data for **9i**: colorless solid (58% yield), m.p. 65–67 °C; ^1^H NMR (CDCl_3_) *δ* 10.36 (s, 1H), 8.10 (d, *J* = 8.42 Hz, 2H), 7.83 (s, 2H), 7.37 (d, *J* = 8.42 Hz, 2H), 6.80–7.06 (m, 3H), 4.84 (d, *J* = 6.02 Hz, 2H), 4.50 (t, *J* = 5.00 Hz, 2H), 4.28 (t, *J* = 5.00 Hz, 2H), 2.69 (s, 3H); ^13^C NMR (CDCl_3_) *δ* 173.13, 168.04, 158.38, 155.89, 154.12, 143.02, 139.57, 135.77, 135.61, 128.17, 119.50, 117.95, 117.58, 117.48, 110.76, 110.54, 104.96, 74.99, 68.61, 62.93, 48.96, 18.35. Anal. Calcd. for C_22_H_19_F_2_N_5_O_3_S: C 56.04; H 4.06; N 14.85. Found: C 56.17; H 3.94; N 14.72.

#### 3.1.4. General Approach to Preparation of Title Compounds **10a**–**10o**

Compounds **9** (3 mmol) were dissolved in 30 mL of alkyl alcohol under an ice bath. Subsequently, sodium alkyl alcohol (6 mmol) in alkyl alcohol (15 mL) was added dropwise slowly. The reaction was well stirred under an ice bath for 4-5 h. On completion, the solvent was steamed to generate crude products, which were finally separated through column chromatography and washed by ethyl acetate/petroleum ether (1:6, *v*/*v*) to produce the designed compounds **10a**–**10o**, and corresponding spectral data of compounds **10a**–**10o** are provided below. ^1^H NMR and ^13^C NMR spectra are depicted in Supplementary Materials. 

Data for **10a**: colorless solid (64% yield), m.p. 76–78 °C; ^1^H NMR (CDCl_3_) *δ* 9.75 (s, 1H), 7.93 (d, *J* = 2.40 Hz, 1H), 7.69–7.74 (m, 3H), 7.31 (d, *J* = 8.42 Hz, 2H), 6.49 (t, *J* = 2.00 Hz, 1H), 4.30–4.52 (m, 4H), 4.24 (s, 3H), 3.64 (t, *J* = 5.20 Hz, 2H), 3.41 (s, 3H); ^13^C NMR (CDCl_3_) *δ* 172.04, 169.58, 141.30, 139.81, 134.73, 128.33, 126.72, 119.54, 117.76, 107.88, 70.38, 63.82, 61.53, 59.28, 44.58. Anal. Calcd. for C_18_H_20_N_4_O_4_: C 60.66; H 5.66; N 15.72. Found: C 60.52; H 5.81; N 15.85.

Data for **10b**: colorless solid (62% yield), m.p. 68–70 °C; ^1^H NMR (CDCl_3_) *δ* 9.78 (s, 1H), 7.93 (d, *J* = 2.40 Hz, 1H), 7.69–7.75 (m, 3H), 7.31 (d, *J* = 8.42 Hz, 2H), 6.49 (t, *J* = 2.40 Hz, 1H), 4.61 (q, *J* = 6.80 Hz, 2H), 4.53 (d, *J* = 6.00 Hz, 2H), 4.31 (t, *J* = 5.00 Hz, 2H), 3.66 (t, *J* = 5.00 Hz, 2H), 3.41 (s, 3H), 1.35 (t, *J* = 7.20 Hz, 3H); ^13^C NMR (CDCl_3_) *δ* 171.41, 169.63, 141.21, 139.69, 134.96, 128.28, 126.80, 119.56, 117.97, 107.88, 71.06, 70.40, 63.77, 59.86, 59.28, 44.53, 15.35. Anal. Calcd. for C_19_H_22_N_4_O_4_: C 61.61; H 5.99; N 15.13. Found: C 61.48; H 5.87; N 15.26.

Data for **10c**: colorless solid (61% yield), m.p. 56–58 °C; ^1^H NMR (CDCl_3_) *δ* 9.78 (s, 1H), 7.94 (d, *J* = 2.40 Hz, 1H), 7.69–7.75 (m, 3H), 7.34 (d, *J* = 8.42 Hz, 2H), 6.49 (d, *J* = 2.00 Hz, 1H), 4.50–4.54 (m, 4H), 4.31 (t, *J* = 5.20 Hz, 2H), 3.66 (t, *J* = 5.20 Hz, 2H), 3.41 (s, 3H), 1.70–1.77 (m, 2H), 0.97 (t, *J* = 7.60 Hz, 3H); ^13^C NMR (CDCl_3_) *δ* 171.41,169.69, 141.27, 139.77, 134.83, 128.21, 119.54, 118.07, 107.87, 76.45, 70.41, 63.76, 59.28, 53.45, 44.48, 23.14, 10.09. Anal. Calcd. for C_20_H_24_N_4_O_4_: C 62.49; H 6.29; N 14.57. Found: C 62.64; H 6.15; N 14.46.

Data for **10d**: yellow solid (60% yield), m.p. 66–68 °C; ^1^H NMR (CDCl_3_) *δ* 9.78 (s, 1H), 7.93 (d, *J* = 2.40 Hz, 1H), 7.69–7.74 (m, 3H), 7.33 (d, *J* = 8.42 Hz, 2H), 6.49 (t, *J* = 2.00 Hz, 1H), 4.52–4.58 (m, 4H), 4.31 (t, *J* = 5.20 Hz, 2H), 3.65 (t, *J* =4.80 Hz, 2H), 3.41 (s, 3H), 1.37–1.73 (m, 4H), 0.93 (t, *J* = 7.60 Hz, 3H); ^13^C NMR (CDCl_3_) *δ* 171.43, 169.69, 141.25, 139.75, 134.87, 128.22, 126.75, 119.54, 118.05, 107.87, 74.79, 70.41, 63.75, 59.28, 44.49, 31.70, 18.75, 13.63. Anal. Calcd. for C_21_H_26_N_4_O_4_: C 63.30; H 6.58; N 14.06. Found: C 63.17; H 6.73; N 14.19.

Data for **10e**: colorless solid (64% yield), m.p. 75–77 °C; ^1^H NMR (CDCl_3_) *δ* 9.75 (s, 1H), 7.92 (d, *J* = 2.40 Hz, 1H), 7.69–7.72 (m, 3H), 7.31 (d, *J* = 8.42 Hz, 2H), 6.48 (t, *J* = 2.00 Hz, 1H), 4.29–4.52 (m, 4H), 4.23 (s, 3H), 3.55–3.71 (m, 4H), 1.22 (t, *J* = 6.80 Hz, 3H); ^13^C NMR (CDCl_3_) *δ* 172.11, 169.61, 141.32, 139.86, 134.79, 128.33, 126.72, 119.58, 117.73, 107.89, 68.19, 66.87, 64.07, 61.54, 59.42, 44.60, 15.20. Anal. Calcd. for C_19_H_22_N_4_O_4_: C 61.61; H 5.99; N 15.13. Found: C 61.73; H 5.85; N 15.01.

Data for **10f**: yellow solid (63% yield), m.p. 97–99 °C; ^1^H NMR (CDCl_3_) *δ* 9.78 (s, 1H), 7.93 (d, *J* = 2.40 Hz, 1H), 7.69–7.73 (m, 3H), 7.31 (d, *J* = 8.42 Hz, 2H), 6.48 (t, *J* = 2.40 Hz, 1H), 4.52–4.63 (m, 4H), 4.30 (t, *J* = 5.20 Hz, 2H), 3.55–3.70 (m, 4H), 1.35 (t, *J* = 6.80 Hz, 3H), 1.22 (t, *J* = 6.80 Hz, 3H); ^13^C NMR (CDCl_3_) *δ* 171.46, 169.64, 141.30, 139.82, 134.93, 128.24, 126.70, 119.53, 117.93, 107.86, 71.06, 68.19, 66.86, 64.00, 59.92, 44.54, 15.34, 15.18. Anal. Calcd. for C_20_H_24_N_4_O_4_: C 62.49; H 6.29; N 14.57. Found: C 62.34; H 6.43; N 14.70.

Data for **10g**: colorless solid (60% yield), m.p. 57–59 °C; ^1^H NMR (CDCl_3_) *δ* 9.79 (s, 1H), 7.94 (d, *J* = 2.40 Hz, 1H), 7.69–7.75 (m, 3H), 7.34 (d, *J* =8.42Hz, 2H), 6.49 (t, *J* =2.00 Hz, 1H), 4.50–4.55 (m, 4H), 4.30 (t, *J* = 5.22 Hz, 2H), 3.55–3.71 (m, 4H), 1.72–1.77 (m, 2H), 1.22 (t, *J* = 6.80 Hz, 3H), 0.97 (t, *J* = 7.60 Hz, 3H); ^13^C NMR (CDCl_3_) *δ* 171.42, 169.69, 141.22, 139.70, 134.93, 128.19, 126.80, 119.57, 118.04, 107.88, 76.45, 68.19, 66.85, 63.98, 59.66, 44.46, 23.14, 15.18, 10.09. Anal. Calcd. for C_21_H_26_N_4_O_4_: C 63.30; H 6.58; N 14.06. Found: C 63.42; H 6.45; N 13.96.

Data for **10h**: colorless solid (52% yield), m.p. 99–100 °C; ^1^H NMR (CDCl_3_) *δ* 9.77 (s, 1H), 8.08 (d, *J* = 8.40 Hz, 2H), 7.82 (s, 2H), 7.35 (d, *J* = 8.42 Hz, 2H), 4.53 (d, *J* = 6.00 Hz, 2H), 4.31 (t, *J* = 4.80 Hz, 2H), 4.24 (s, 3H), 3.65 (t, *J* = 4.80 Hz, 2H), 3.41 (s, 3H); ^13^C NMR (CDCl_3_) *δ* 172.06, 169.59, 139.44, 135.96, 135.71, 128.14, 119.38, 117.74, 70.38, 63.84, 61.54, 59.34, 59.28, 44.59. Anal. Calcd. for C_17_H_19_N_5_O_4_: C 57.14; H 5.36; N 19.60. Found: C 57.03; H 5.48; N 19.52.

Data for **10i**: colorless solid (53% yield), m. p. 54–56 °C; ^1^H NMR (CDCl_3_) *δ* 9.81 (s, 1H), 8.09 (d, *J* = 8.80 Hz, 2H), 7.82 (s, 2H), 7.35 (d, *J* = 8.42 Hz, 2H), 4.62 (q, *J* = 6.80 Hz, 2H), 4.54 (d, *J* = 6.00 Hz, 2H), 4.32 (t, *J* = 4.80 Hz, 2H), 3.65 (t, *J* = 4.80 Hz, 2H), 3.41 (s, 3H), 1.35 (t, *J* = 6.80 Hz, 3H); ^13^C NMR (CDCl_3_) *δ* 171.43, 169.63, 139.41, 136.12, 135.71, 128.07, 119.35, 117.95, 71.07, 70.40, 63.78, 59.84, 59.28, 44.54, 15.33. Anal. Calcd. for C_18_H_21_N_5_O_4_: C 58.21; H 5.70; N 18.86. Found: C 58.33; H 5.59; N 18.97.

Data for **10j**: colorless solid (49% yield), m.p. 57–59 °C; ^1^H NMR (CDCl_3_) *δ* 9.81 (s, 1H), 8.08 (d, *J* = 8.40 Hz, 2H), 7.83 (s, 2H), 7.35 (d, *J* = 8.82 Hz, 2H), 4.51–4.57 (m, 4H), 4.31 (t, *J* = 4.80 Hz, 2H), 3.65 (t, *J* = 4.80 Hz, 2H), 3.42 (s, 3H), 1.71–1.76 (m, 2H), 0.96 (t, *J* = 7.60 Hz, 3H); ^13^C NMR (CDCl_3_) *δ* 171.43, 169.68, 139.42, 136.06, 135.70, 128.01, 119.36, 118.03, 76.46, 70.41, 63.77, 59.65, 59.28, 44.48, 23.13, 10.08. Anal. Calcd. for C_19_H_23_N_5_O_4_: C 59.21; H 6.02; N 18.17. Found: C 59.08; H 6.14; N 18.06.

Data for **10k**: colorless solid (52% yield), m.p. 47–49 °C; ^1^H NMR (CDCl_3_) *δ* 9.81 (s, 1H), 8.07–8.10 (m, 2H), 7.83 (s, 2H), 7.34 (d, *J* = 8.82 Hz, 2H), 4.54–4.58 (m, 4H), 4.31–4.33 (m, 2H), 3.65–3.67 (m, 2H), 3.42 (s, 3H), 1.65–1.72 (m, 2H), 1.36–1.42 (m, 2H), 0.92 (t, *J* = 6.80 Hz, 3H); ^13^C NMR (CDCl_3_) *δ* 171.46, 169.69, 139.42, 136.08, 135.70, 128.01, 119.36, 118.04, 74.81, 70.41, 63.78, 59.65, 59.28, 44.50, 31.68, 18.74, 13.62. Anal. Calcd. for C_20_H_25_N_5_O_4_: C 60.14; H 6.31; N 17.53. Found: C 60.05; H 6.43; N 17.65.

Data for **10l**: colorless solid (57% yield), m.p. 48–50 °C; ^1^H NMR (CDCl_3_) *δ* 9.81 (s, 1H), 8.07 (d, *J* = 8.38 Hz, 2H), 7.83 (s, 2H), 7.34 (d, *J* = 8.42 Hz, 2H), 4.53–4.57 (m, 4H), 4.31 (t, *J* = 4.80 Hz, 2H), 3.64 (t, *J* = 5.20 Hz, 2H), 3.42 (s, 3H), 1.66–1.71 (m, 2H), 1.30–1.34 (m, 4H), 0.87 (t, *J* = 6.80 Hz, 3H); ^13^C NMR (CDCl_3_) *δ* 171.45, 169.70, 139.42, 136.10, 135.70, 128.00, 119.35, 75.09, 70.42, 63.78, 59.30, 44.50, 29.39, 27.56, 22.19, 13.88. Anal. Calcd. for C_21_H_27_N_5_O_4_: C 61.00; H 6.58; N 16.94. Found: C 61.13; H 6.70; N 16.81.

Data for **10m**: colorless solid (51% yield), m.p. 78–79 °C; ^1^H NMR (CDCl_3_) *δ* 9.78 (s, 1H), 8.08 (d, *J* = 8.42 Hz, 2H), 7.82 (s, 2H), 7.34 (d, *J* = 8.42 Hz, 2H), 4.53 (d, *J* = 6.00 Hz, 2H), 4.30 (t, *J* = 5.20 Hz, 2H), 4.23 (s, 3H), 3.55–3.78 (m, 4H), 1.22 (t, *J* = 6.80 Hz, 3H); ^13^C NMR (CDCl_3_) *δ* 172.09, 169.59, 139.45, 136.00, 135.72, 128.12, 119.39, 117.72, 68.17, 66.86, 64.07, 61.54, 59.40, 44.58, 15.18. Anal. Calcd. for C_18_H_21_N_5_O_4_: C 58.21; H 5.70; N 18.86. Found: C 58.12; H 5.82; N 18.97.

Data for **10n**: colorless solid (54% yield), m.p. 69–71 °C; ^1^H NMR (CDCl_3_) *δ* 9.81 (s, 1H), 8.07 (d, *J* = 8.42 Hz, 2H), 7.82 (s, 2H), 7.35 (d, *J* = 8.42 Hz, 2H), 4.54–4.64 (m, 4H), 4.30 (t, *J* = 5.20 Hz, 2H), 3.55–3.71 (m, 4H), 1.35 (t, *J* = 6.80 Hz, 3H), 1.22 (t, *J* = 6.80 Hz, 3H); ^13^C NMR (CDCl_3_) *δ* 171.45, 169.63, 139.41, 136.16, 135.70, 128.05, 119.36, 117.96, 71.08, 68.19, 66.86, 64.01, 59.90, 44.53, 15.33, 15.18. Anal. Calcd. for C_19_H_23_N_5_O_4_: C 59.21; H 6.02; N 18.17. Found: C 59.15; H 6.12; N 18.06.

Data for **10o**: colorless solid (55% yield), m.p. 72–74 °C; ^1^H NMR (CDCl_3_) *δ* 9.82 (s, 1H), 8.07–8.10 (m, 2H), 7.83 (s, 2H), 7.34 (d, *J* = 8.42 Hz, 2H), 4.50–4.56 (m, 4H), 4.31 (t, *J* = 5.20 Hz, 2H), 3.55–3.71 (m, 4H), 1.71–1.76 (m, 2H), 1.22 (t, *J* = 7.00 Hz, 3H), 0.97 (t, *J* = 7.00 Hz, 3H); ^13^C NMR (CDCl_3_) *δ* 171.45, 169.69, 139.42, 136.09, 135.70, 127.99, 119.36, 118.02, 76.45, 68.19, 66.86, 64.00, 59.71, 44.47, 23.13, 15.19, 10.08. Anal. Calcd. for C_20_H_25_N_5_O_4_: C 60.14; H 6.31; N 17.53. Found: C 60.27; H 6.19; N 17.41.

### 3.2. Biological Activity Test

All bioassays were accomplished on six kinds of typical weeds cultured in the laboratory. The herbicidal activities against *Brassica juncea*, *Chenopodium serotinum*, *Rumex acetosa*, *Alopecurus aequalis*, *Polypogon fugax,* and *Poa annua* of designed compounds were measured using a pot culture method [33]. First, tested compounds were dissolved with DMF, including 0.1% Tween-80 emulsifying agent, which was subsequently diluted by water to the required doses of 1500 and 150 g/ha. Flowerpots with a diameter of 7.5 cm were plated with composite soil, and then the seeds of six weeds were sowed at 25 °C in the greenhouse. When the weeds grew to their three-leaf stage, each title compound was sprayed by post-emergence application. After air-drying, the weeds were kept in the greenhouse for normal cultivation at 25 °C. A mixture of an equivalent quantity of DMF, Tween-80, and water was sprayed as a negative contrast. Additionally, NK-9717 was used as a positive control. The fresh weight of the aforementioned ground tissues was surveyed 25 days after post-emergence treatment. The inhibition effect of each compound on the growth of the above weeds was assessed by the percentage change in the weed weight compared to that of the control, as 0% (no effect) and 100% (complete death). Each assessment was repeated in triplicate, and the results were averaged. 

## 4. Conclusions

In summary, twenty-four 2-cyanoacrylate derivatives, including pyrazolyl or 1,2,3-triazolyl unit, were synthesized and screened the herbicidal properties against six weeds. Bioassay results demonstrated that some target compounds showed wonderful herbicidal activities against *Brassica juncea* and *Chenopodium serotinum* at dosages of 1500 and 150 g/ha, and some title compounds expressed moderate to satisfactory herbicidal activities towards *Rumex acetosa*, *Alopecurus aequalis*, *Polypogon fugax,* and *Poa annua* at 1500 g/ha. Among them, compounds **9g**, **10i**, and **10n** presented a wide range of herbicidal activities towards all six weeds. These compounds can be selected as lead pesticides for further structural improvement and bioactivity optimization. 

## Figures and Tables

**Table 1:** Herbicidal activity(inhibition rate, %) of title compounds at 1500 g/ha.

Compd.	Dose	*Brassica* *juncea*	*Chenopodium* *serotinum*	*Rumex* *acetosa*	*Alopecurus* *aequalis*	*Polypogon* *fugax*	*Poa* *annua*
	(g/ha)						
**9a**	1500	100	100	100	45	40	30
**9b**	1500	0	0	0	0	0	0
**9c**	1500	0	0	0	0	0	0
**9d**	1500	100	100	100	50	40	30
**9e**	1500	30	0	0	0	0	0
**9f**	1500	90	90	30	30	30	20
**9g**	1500	100	100	80	70	50	30
**9h**	1500	90	90	0	50	30	30
**9i**	1500	95	100	20	70	40	30
**10a**	1500	100	100	80	80	70	30
**10b**	1500	100	80	50	30	30	30
**10c**	1500	0	0	0	0	0	0
**10d**	1500	0	0	0	0	0	0
**10e**	1500	100	100	100	50	45	20
**10f**	1500	60	100	100	0	0	0
**10g**	1500	0	0	0	0	0	0
**10h**	1500	100	100	0	85	80	50
**10i**	1500	85	90	60	70	70	30
**10j**	1500	50	40	0	30	0	0
**10k**	1500	50	80	0	30	0	0
**10l**	1500	100	0	0	0	0	0
**10m**	1500	90	90	30	70	60	40
**10n**	1500	100	100	100	100	100	70
**10o**	1500	100	100	60	90	0	0
**NK-9717**	1500	100	100	100	30	20	10

**Table 2:** Herbicidal activity (inhibition rate, %) of some compounds at 150 g/ha.

Compd.	Dose	*Brassica* *juncea*	*Chenopodium* *serotinum*	*Rumex* *acetosa*	*Alopecurus* *aequalis*	*Polypogon* *fugax*	*Poa* *annua*
	(g/ha)						
**9a**	150	80	20	20	0	0	0
**9d**	150	90	90	70	0	0	0
**9f**	150	80	80	20	20	20	0
**9g**	150	60	80	30	20	20	20
**9h**	150	70	70	0	20	20	20
**9i**	150	85	80	0	0	0	0
**10a**	150	80	60	20	0	0	0
**10b**	150	80	20	30	0	0	0
**10e**	150	80	40	20	0	0	0
**10f**	150	20	50	20	0	0	0
**10h**	150	75	70	0	0	0	0
**10i**	150	70	75	20	30	30	20
**10k**	150	0	30	0	0	0	0
**10l**	150	0	0	0	0	0	0
**10m**	150	75	85	20	0	0	0
**10n**	150	80	80	40	30	30	20
**10o**	150	60	70	40	20	0	0
**NK-9717**	150	80	75	40	0	0	0

## Data Availability

Not applicable.

## Supplementary Materials

The following supporting information can be downloaded at: https://www.mdpi.com/article/10.3390/molecules28073141/s1, ^1^H NMR and ^13^C NMR spectra of compounds **4a**, **4b**, **5a**, **5b**, **9a**–**9i**, and **10a**–**10o**.
