# Supplementary material for: Design, Synthesis, and Biological Activities of Novel 2-Cyanoacrylate Compounds Containing Substituted Pyrazolyl or 1,2,3-Triazolyl Moiety
Source: Molecules. 2023 Mar 31;28(7):3141. doi: 10.3390/molecules28073141 (PMC10096079; doi:10.3390/molecules28073141)
Supplement: Supplementary file 1 [file molecules-28-03141-s001.zip › molecules-2294350-supplementary.pdf]

molecules

MDPI

Supporting Information

Design, Synthesis, and Biological Activities of Novel 2-Cyanoacrylate Compounds Containing Substituted Pyrazolyl or 1,2,3-Triazolyl Moiety

Yang Wang †, Yudie Chen †, Ye Qian, Jia Chen, Xianchao Du, Yujun Shi *, Baolin Xu, Sheng Hua and Hong Dai *

College of Chemistry and Chemical Engineering, Nantong University, Nantong 226019, China; k765786104@163.com (Y.W.); chenyudie2023@163.com (Y.C.); qq1186937868@163.com (Y.Q.); 15642891665@163.com (J.C.); fyuan6586@aliyun.com (X.D.); 19840134319@163.com (B.X.); yaowei265688@126.com (S.H.)

* Correspondence: syj@ntu.edu.cn (Y.S.); dh123@ntu.edu.cn (H.D.); Tel./Fax: +86-513-8501-2851 (Y.S.) ; +86-513-8501-2945 (H.D.)

† These authors contributed equally to this work.

The ^{1}H-NMR and ^{13}C-NMR spectra of compounds **4a**, **4b, 5a**, **5b, 9a–9i** and **10a – 10o** were listed below:

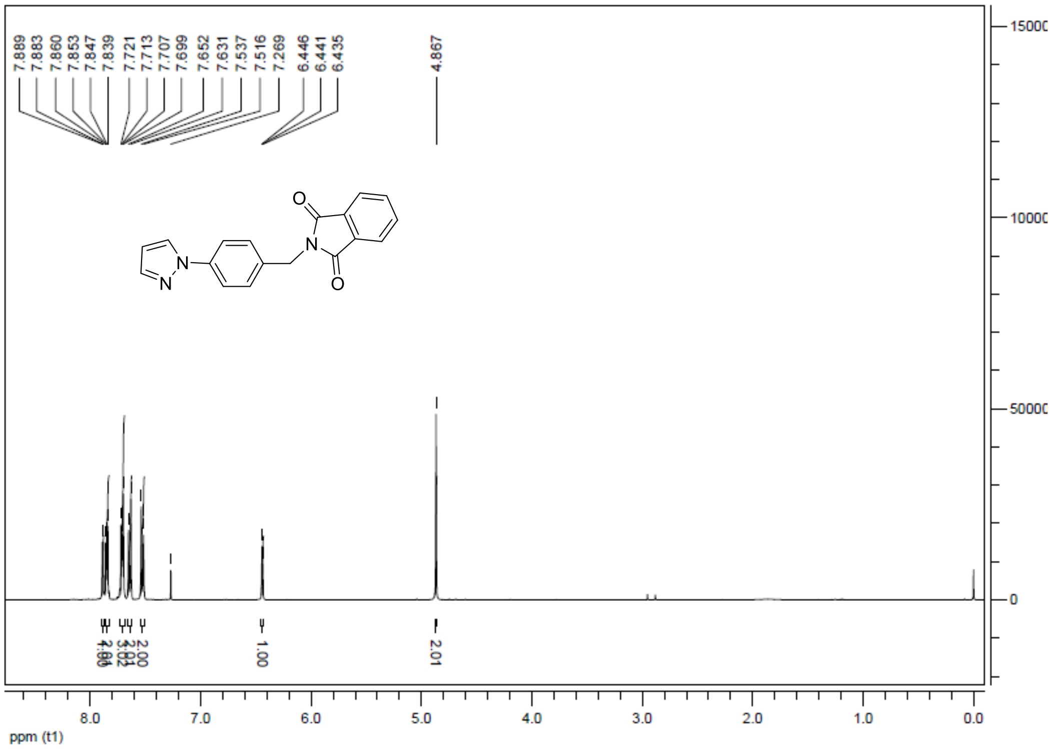

Figure S1. ^{1}H-NMR of compound **4a** (400 MHz, $CDCl_3$)

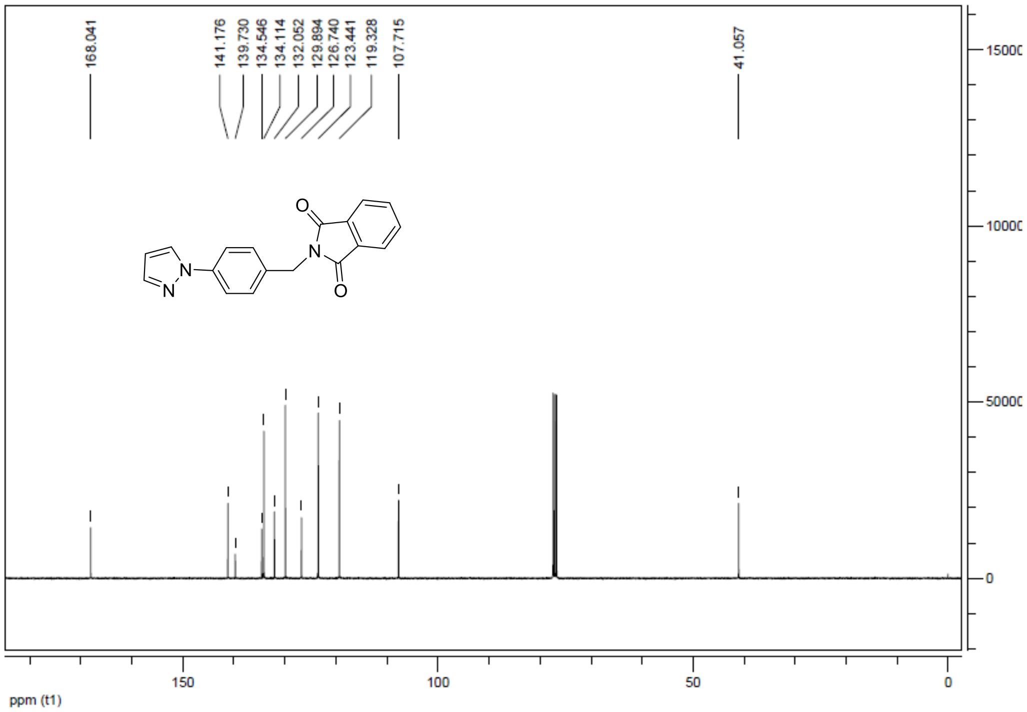

Figure S2. ^{13}C-NMR of compound **4a** (100 MHz, $CDCl_3$)

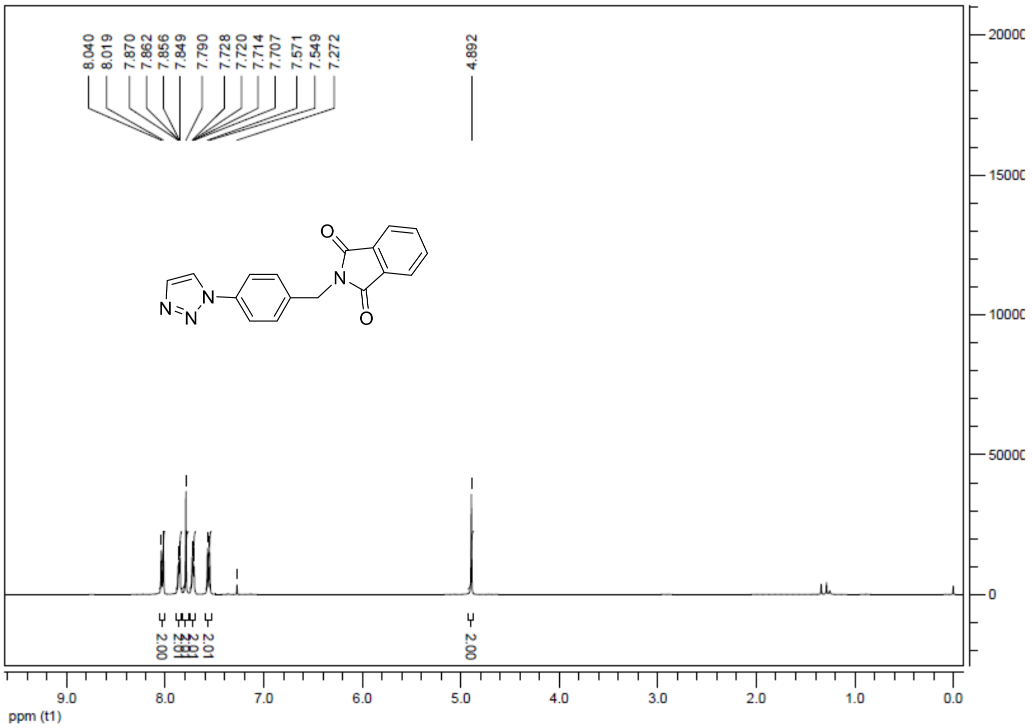

Figure S3. ^{1}H-NMR of compound **4b** (400 MHz, $CDCl_3$)

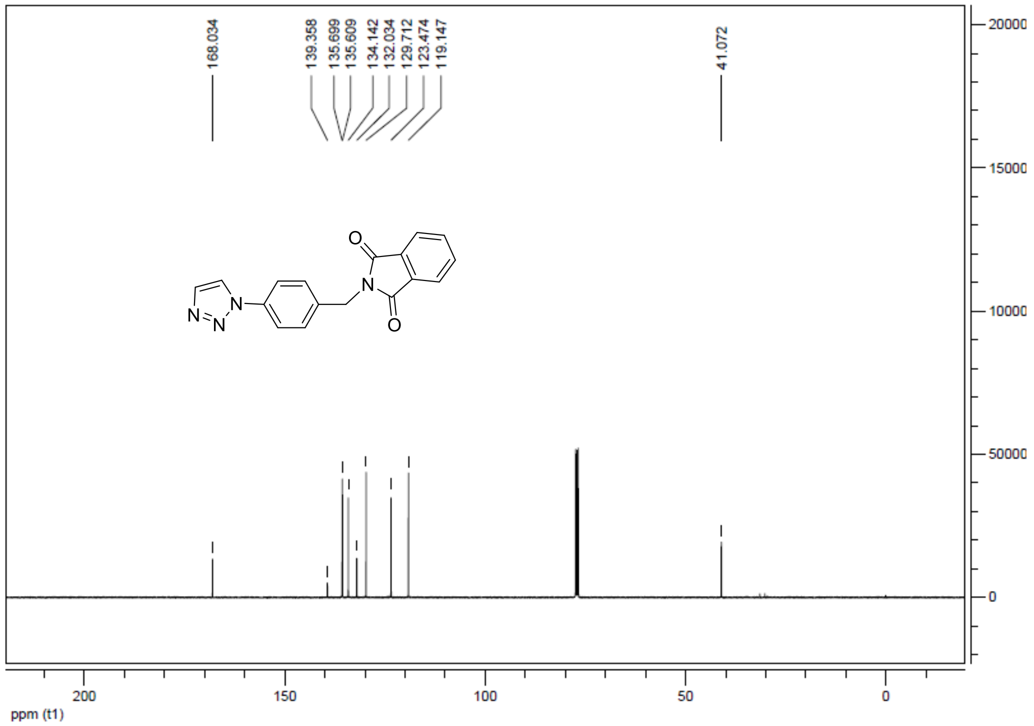

Figure S4. ^{13}C-NMR of compound **4b** (100 MHz, $CDCl_3$)

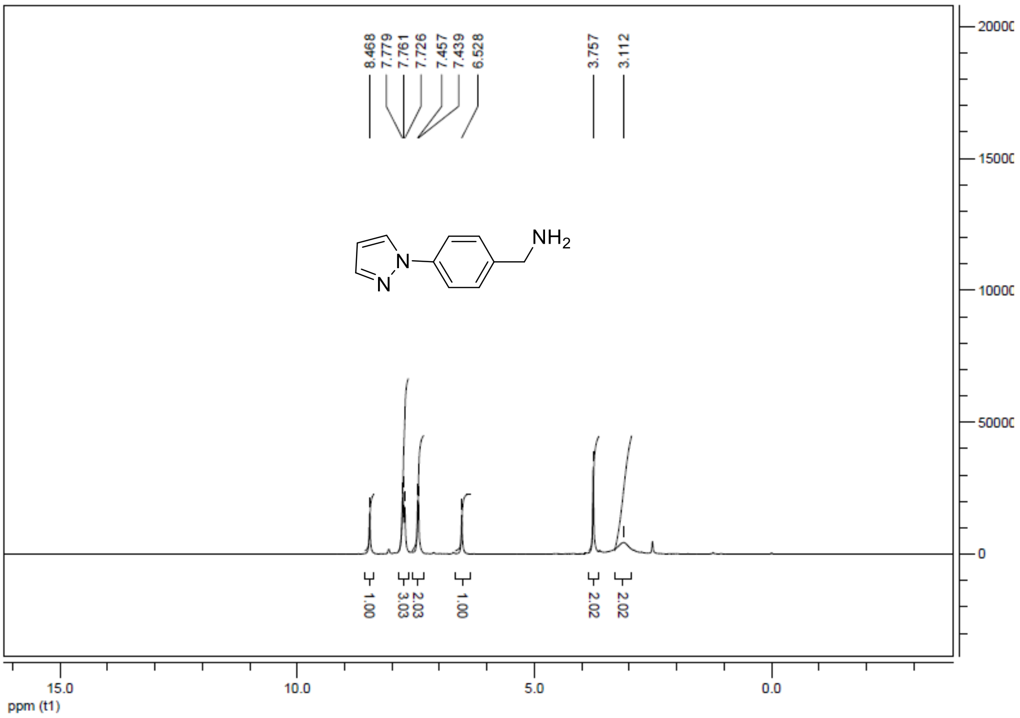

Figure S5. ^{1}H-NMR of compound **5a** (400 MHz, DMSO-d_6)

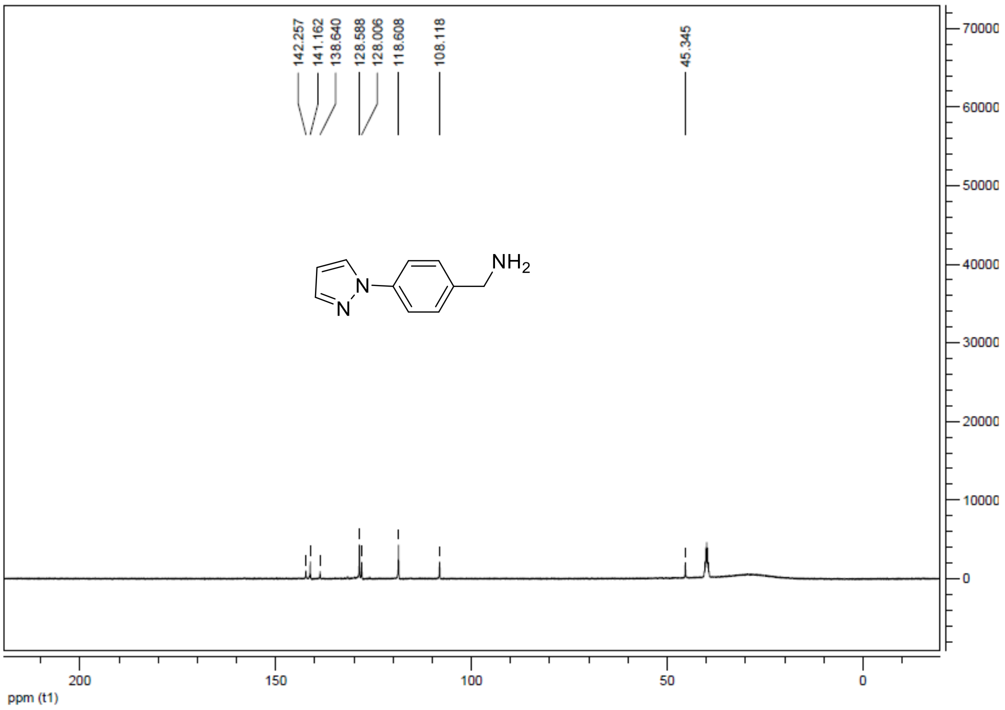

Figure S6. ^{13}C-NMR of compound **5a** (100 MHz, DMSO-d_6)

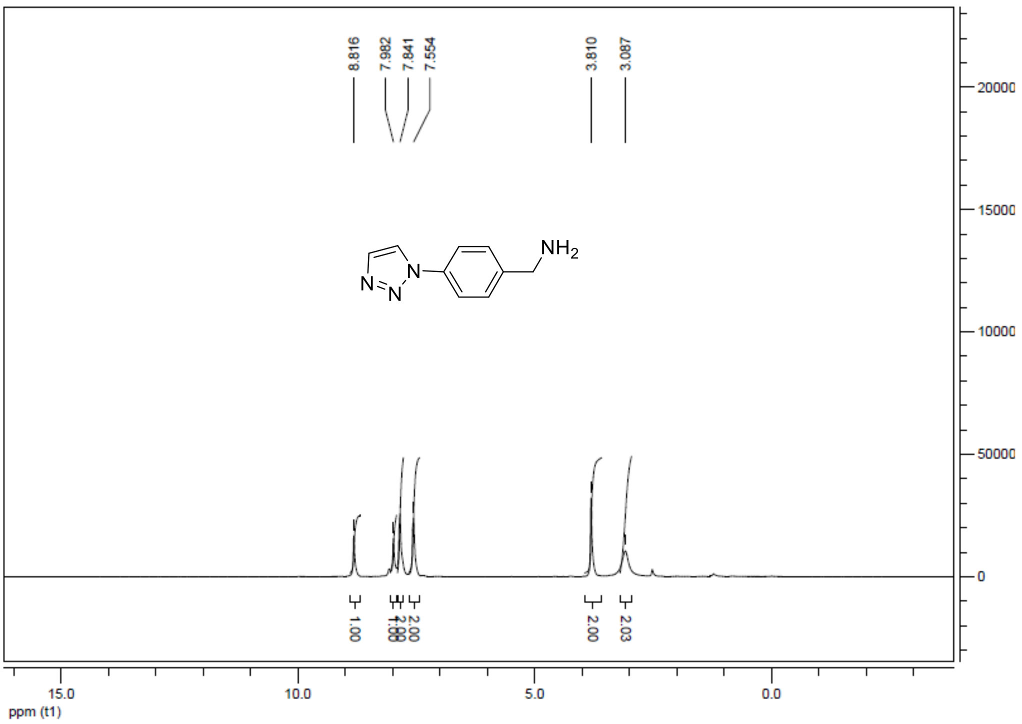

Figure S7. ^{1}H-NMR of compound **5b** (400 MHz, DMSO-d_6)

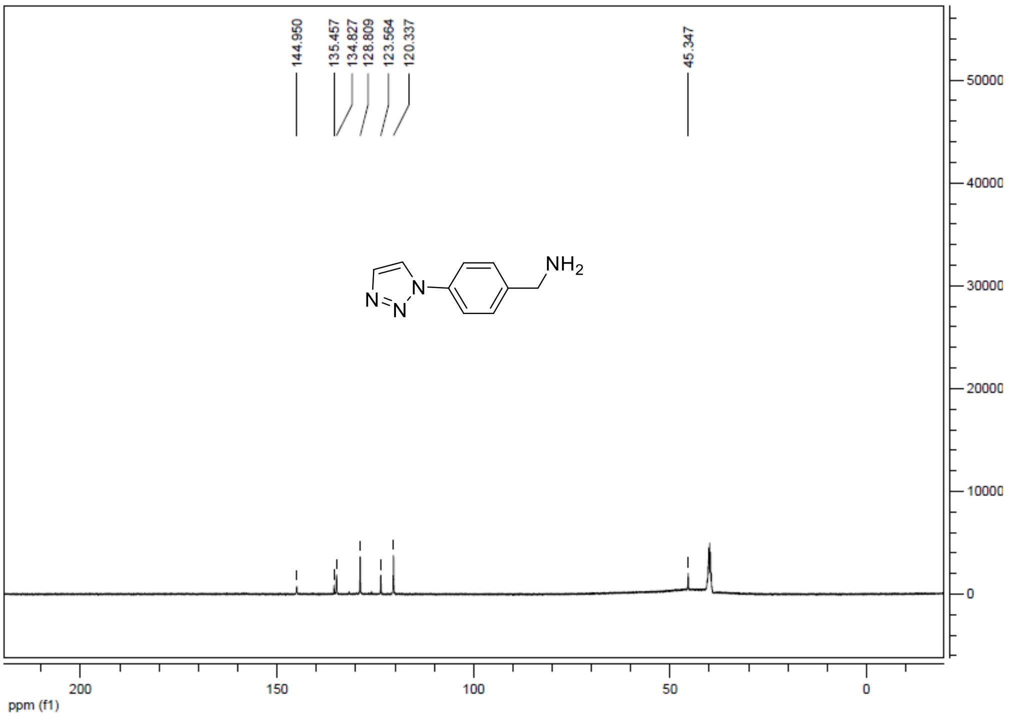

Figure S8. ^{13}C-NMR of compound **5b** (100 MHz, DMSO-d_6)

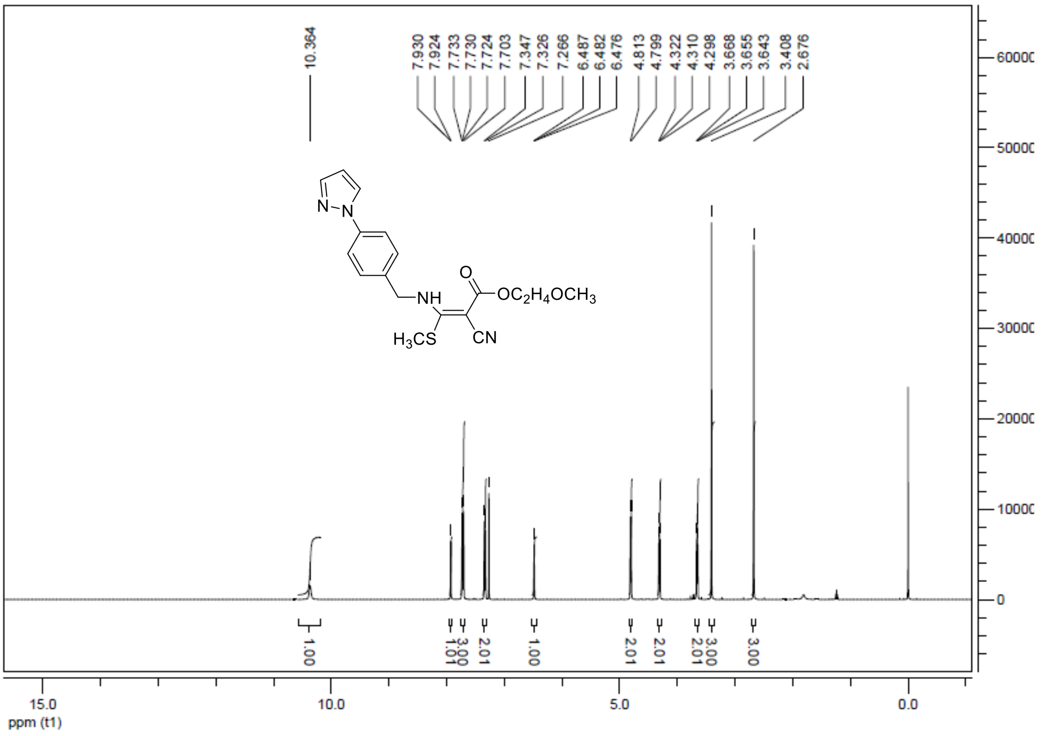

Figure S9. ^{1}H-NMR of compound **9a** (400 MHz, $CDCl_3$)

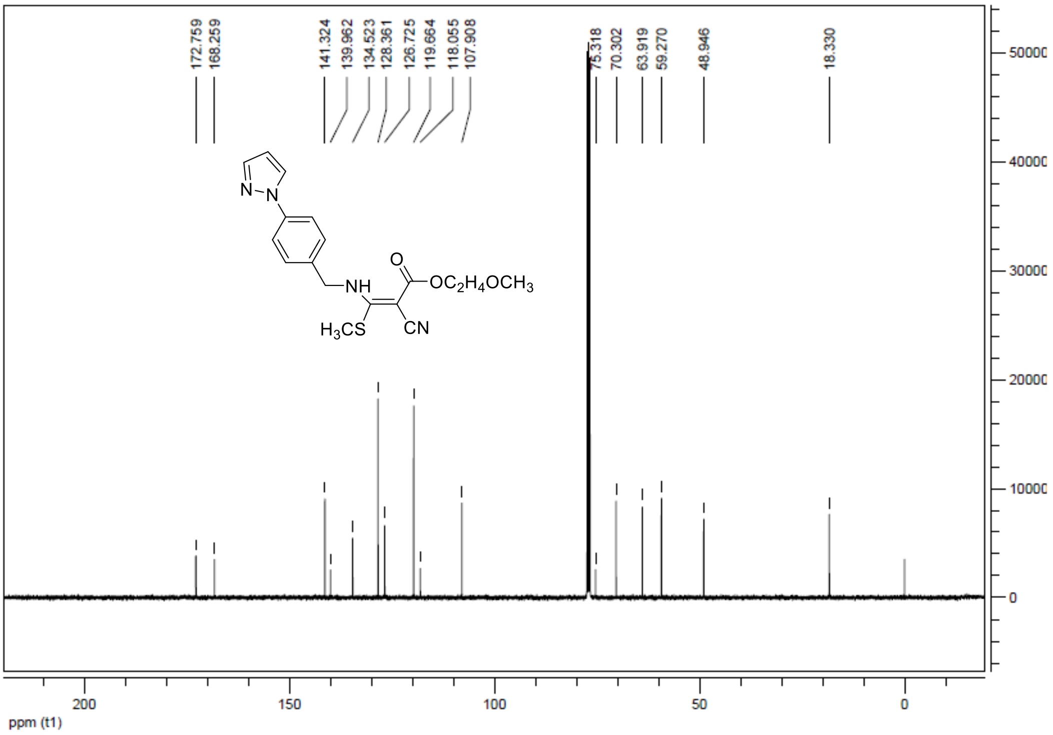

Figure S10. ^{13}C-NMR of compound **9a** (100 MHz, $CDCl_3$)

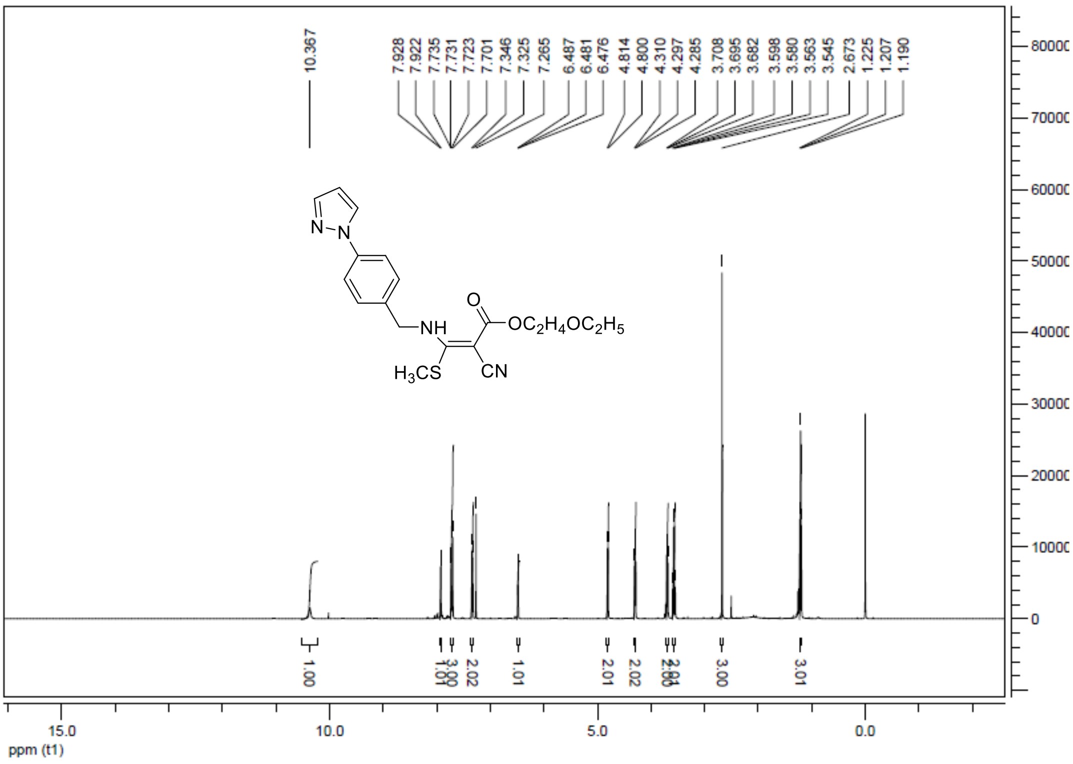

Figure S11. ^{1}H-NMR of compound **9b** (400 MHz, $CDCl_3$).

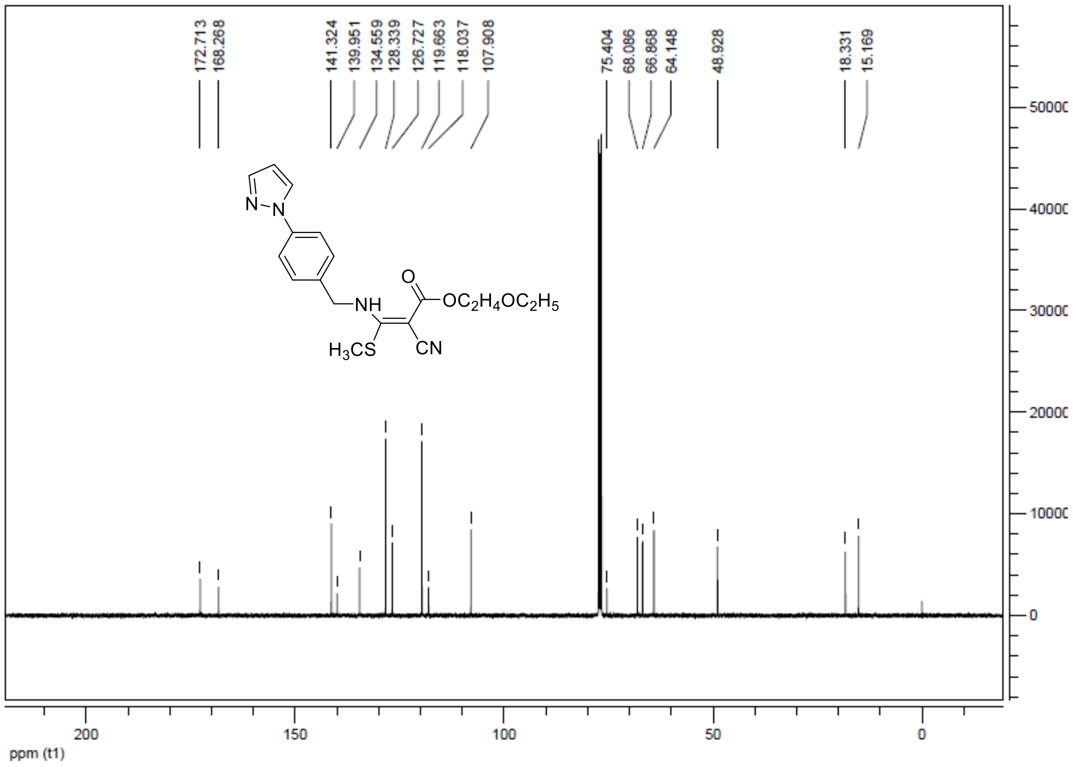

Figure S12. ^{13}C-NMR of compound **9b** (100 MHz, $CDCl_3$)

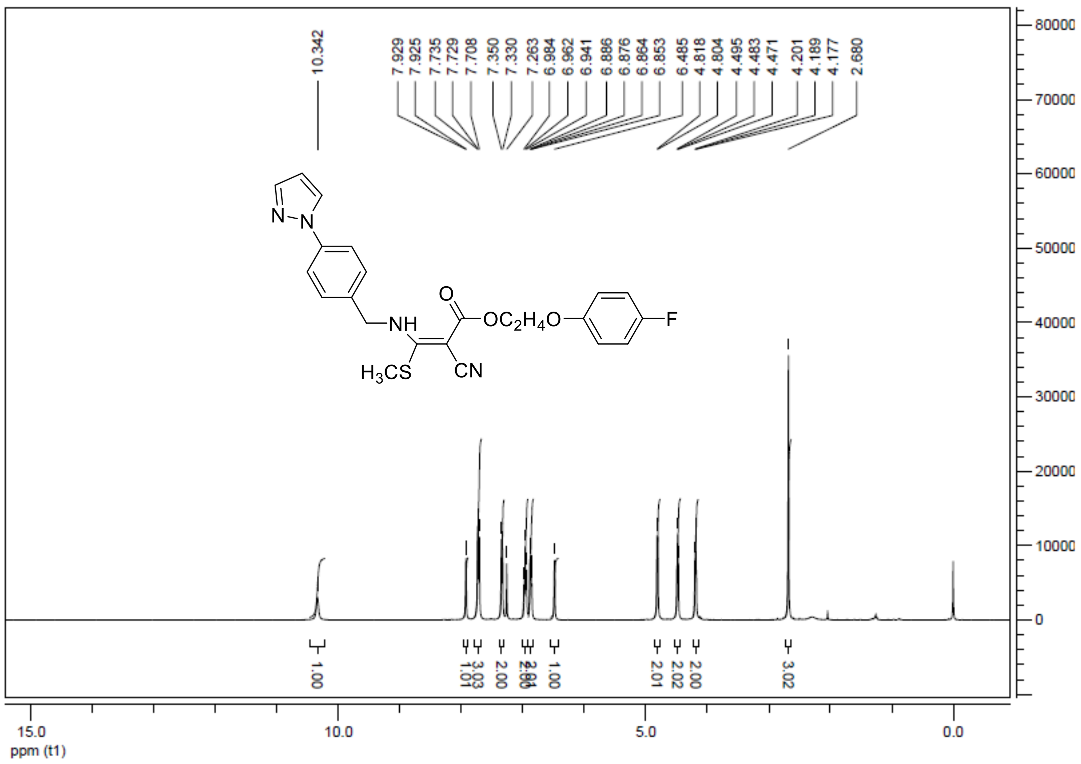

Figure S13. ^{1}H-NMR of compound **9c** (400 MHz, $CDCl_3$)

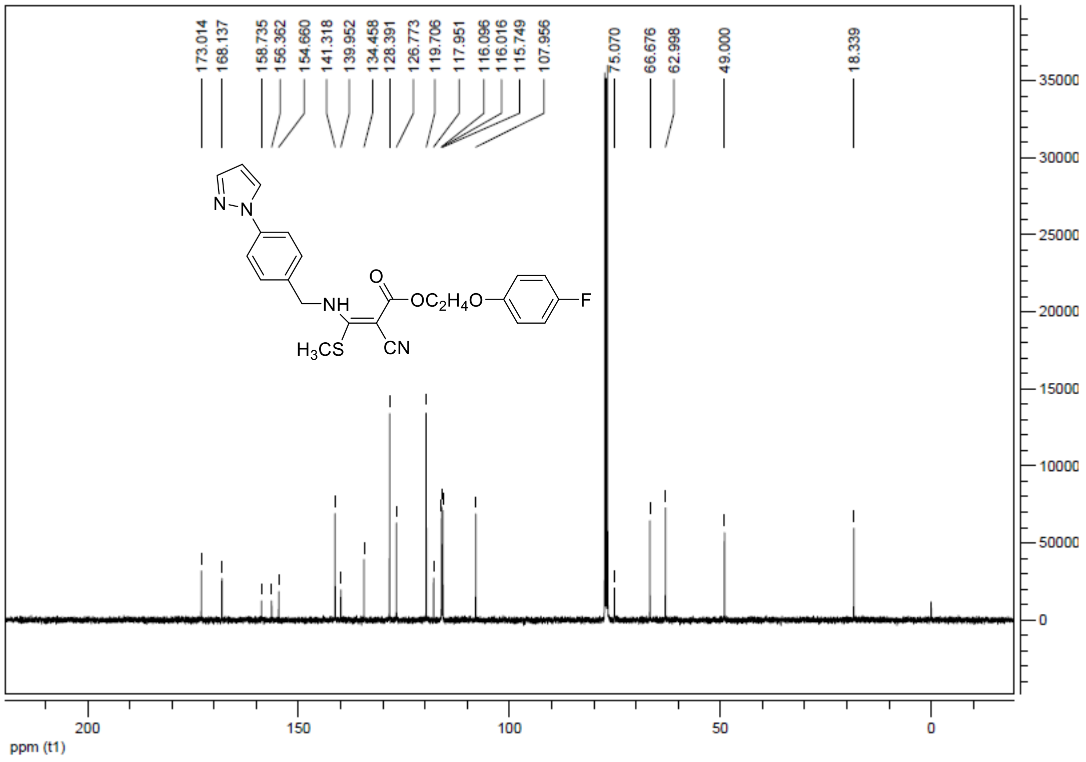

Figure S14. ^{13}C-NMR of compound **9c** (400 MHz, $CDCl_3$)

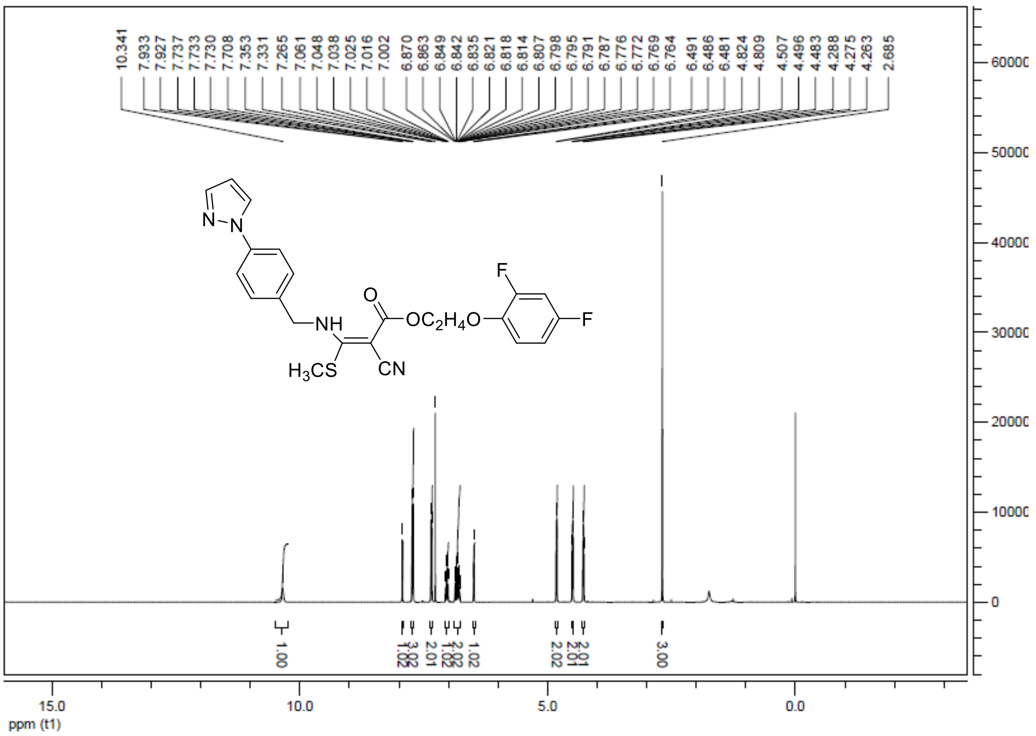

Figure S15. ^{1}H-NMR of compound **9d** (400 MHz, $CDCl_3$)

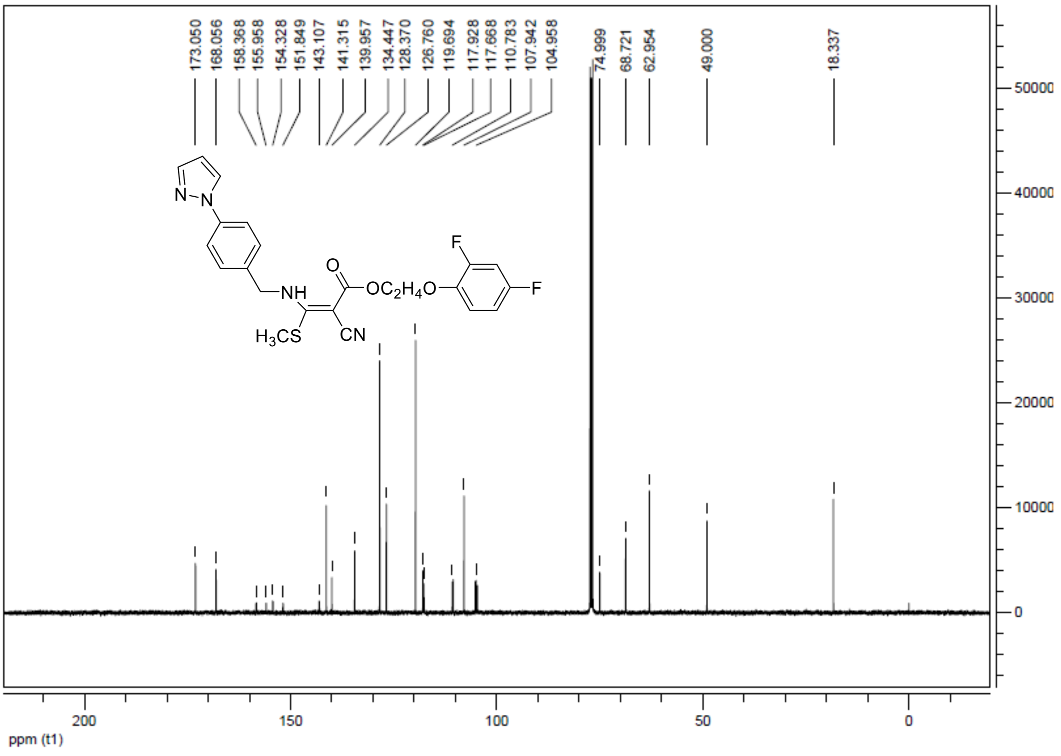

Figure S16. ^{13}C-NMR of compound **9d** (100 MHz, $CDCl_3$)

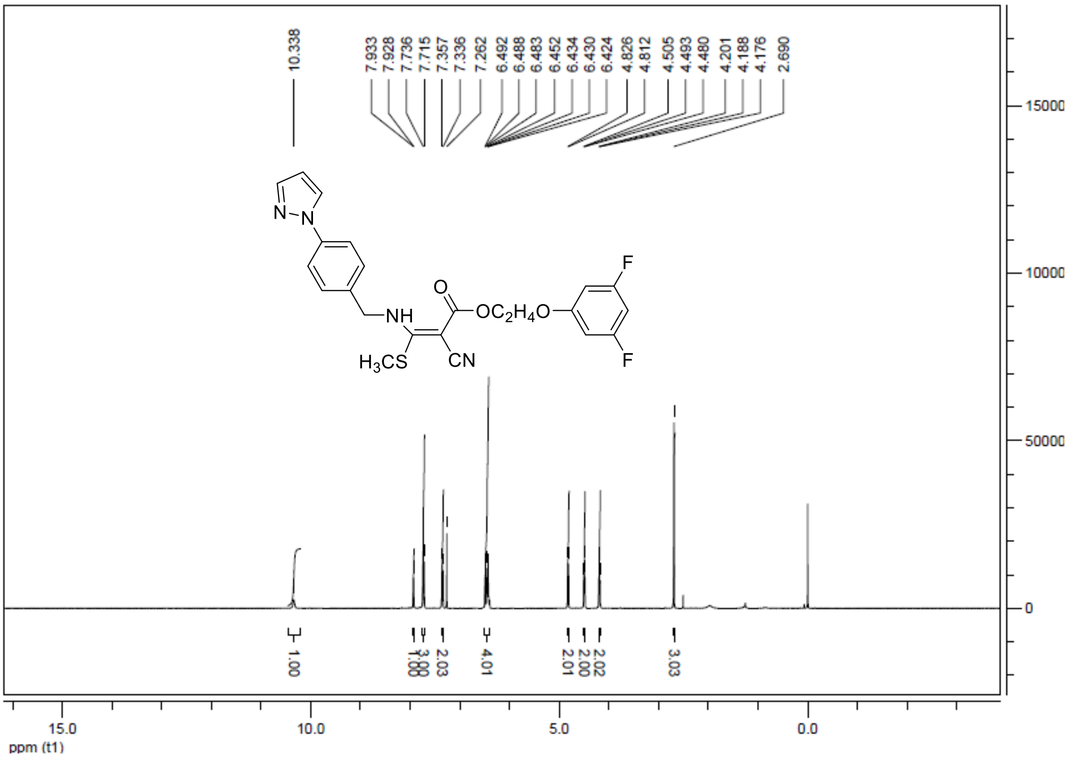

Figure S17. ^{1}H-NMR of compound **9e** (400 MHz, $CDCl_3$)

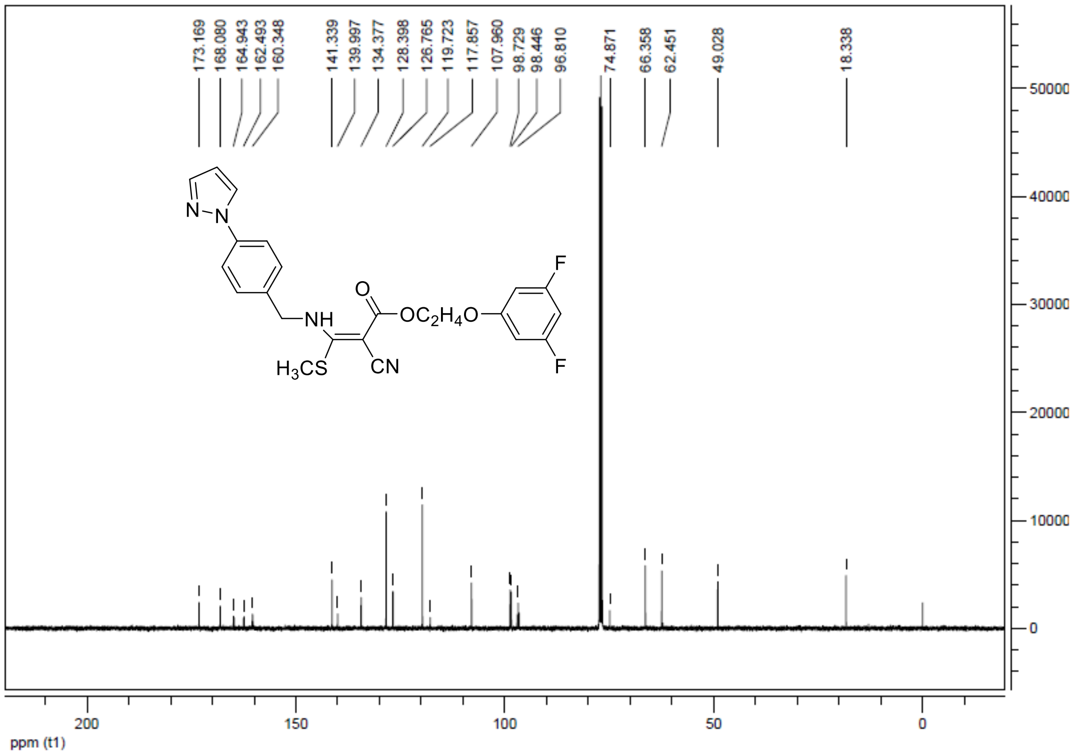

Figure S18. ^{13}C-NMR of compound **9e** (100 MHz, $CDCl_3$)

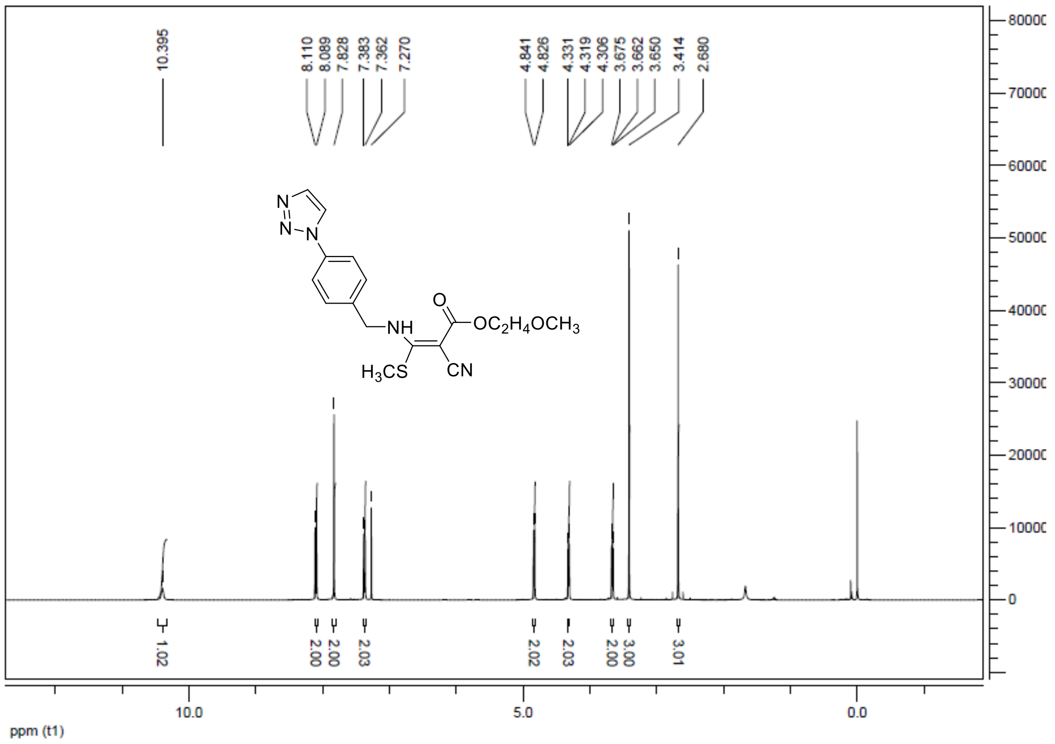

Figure S19. ^{1}H-NMR of compound **9f** (400 MHz, CDCl3)

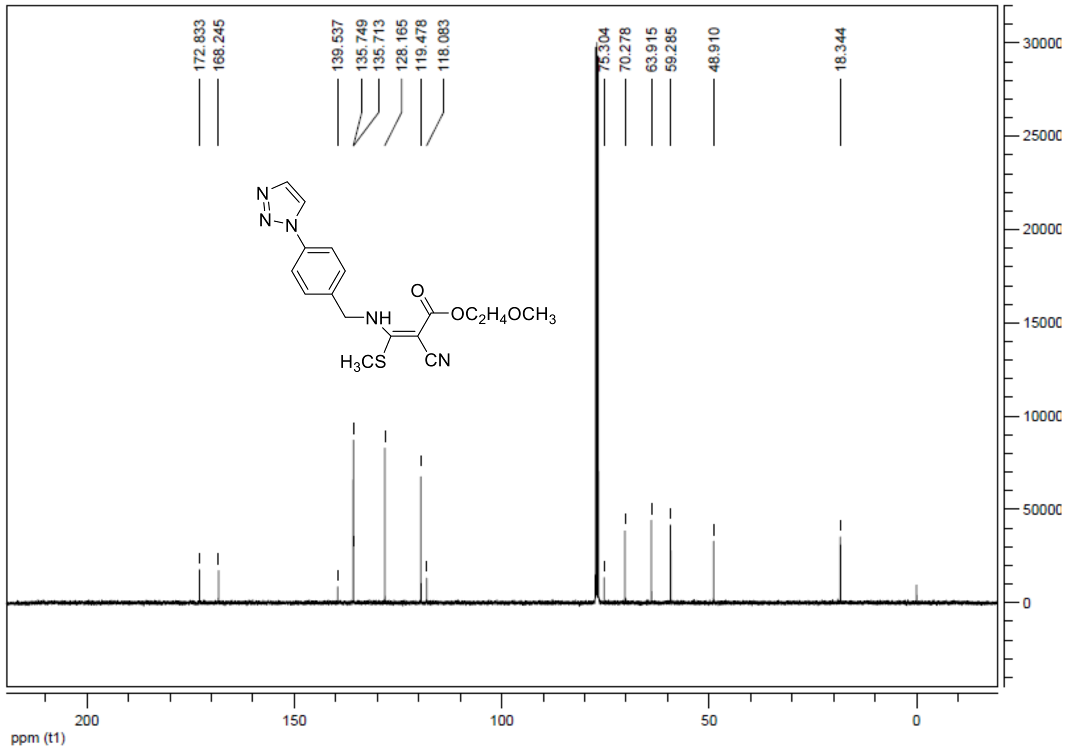

Figure S20. ^{13}C-NMR of compound **9f** (100 MHz, CDCl3)

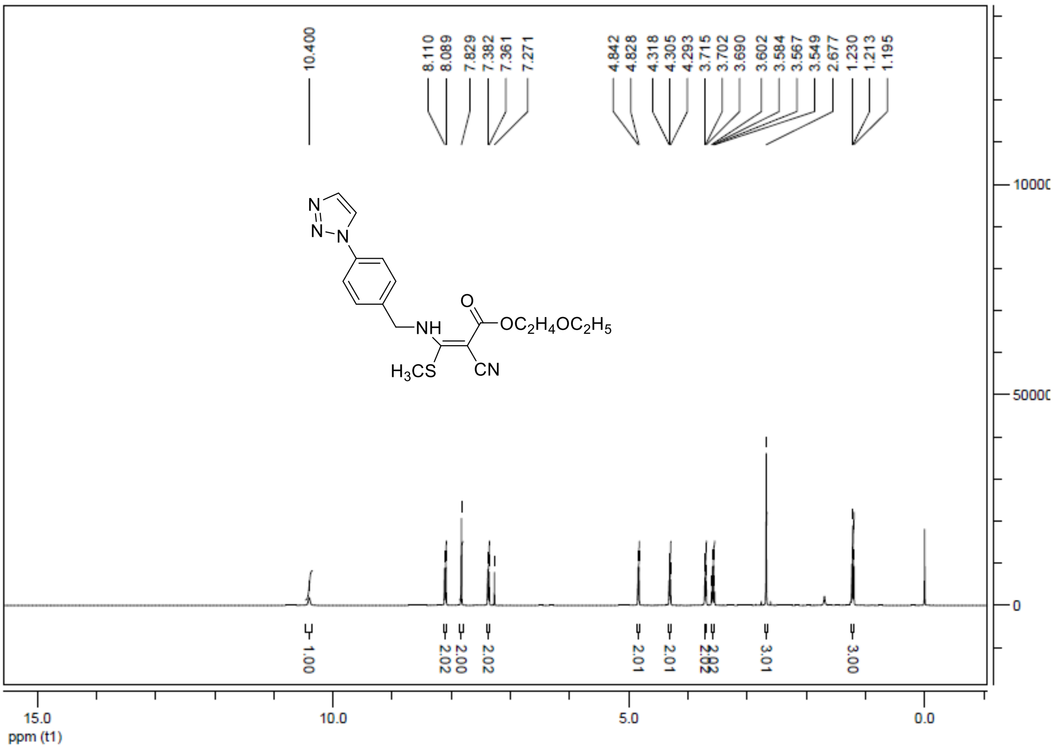

Figure S21. ^{1}H-NMR of compound **9g** (400 MHz, $CDCl_3$)

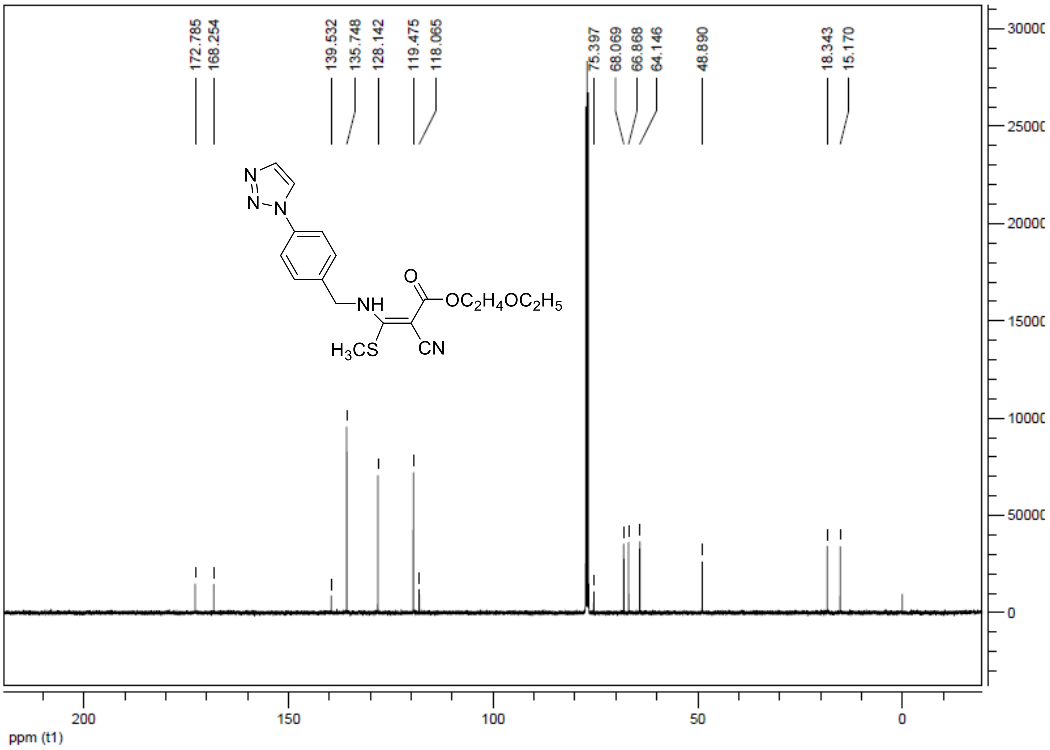

Figure S22. ^{13}C-NMR of compound **9g** (100 MHz, $CDCl_3$)

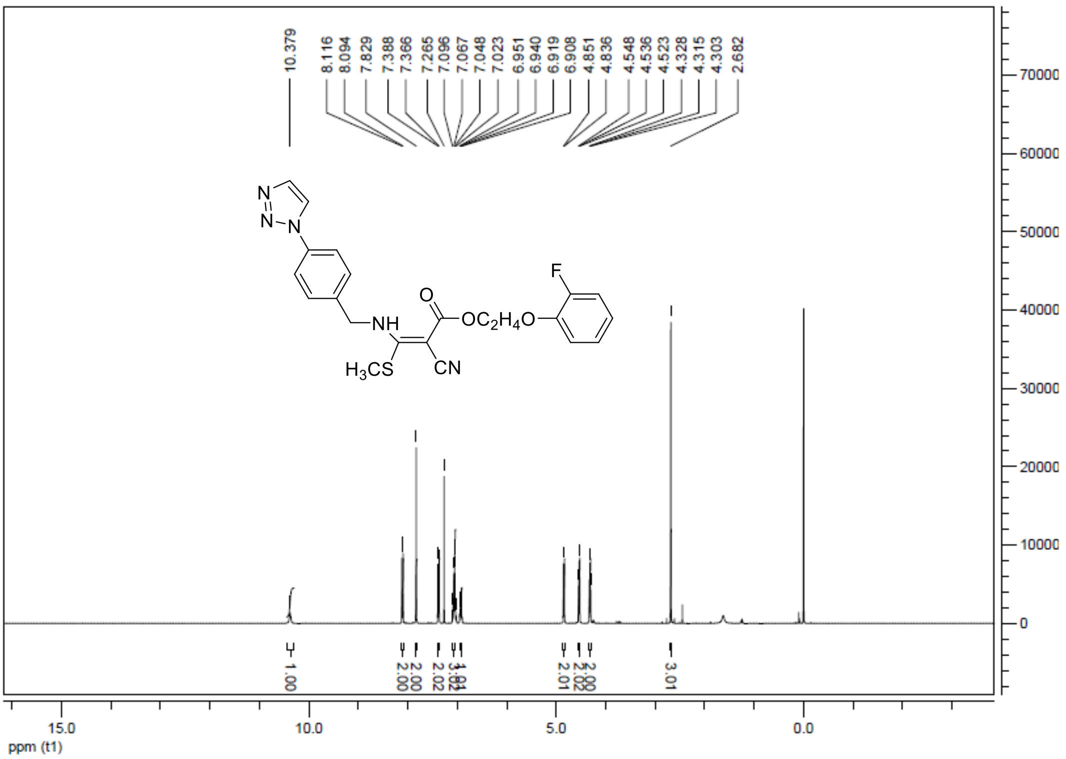

Figure S23. ^{1}H-NMR of compound **9h** (400 MHz, $CDCl_3$)

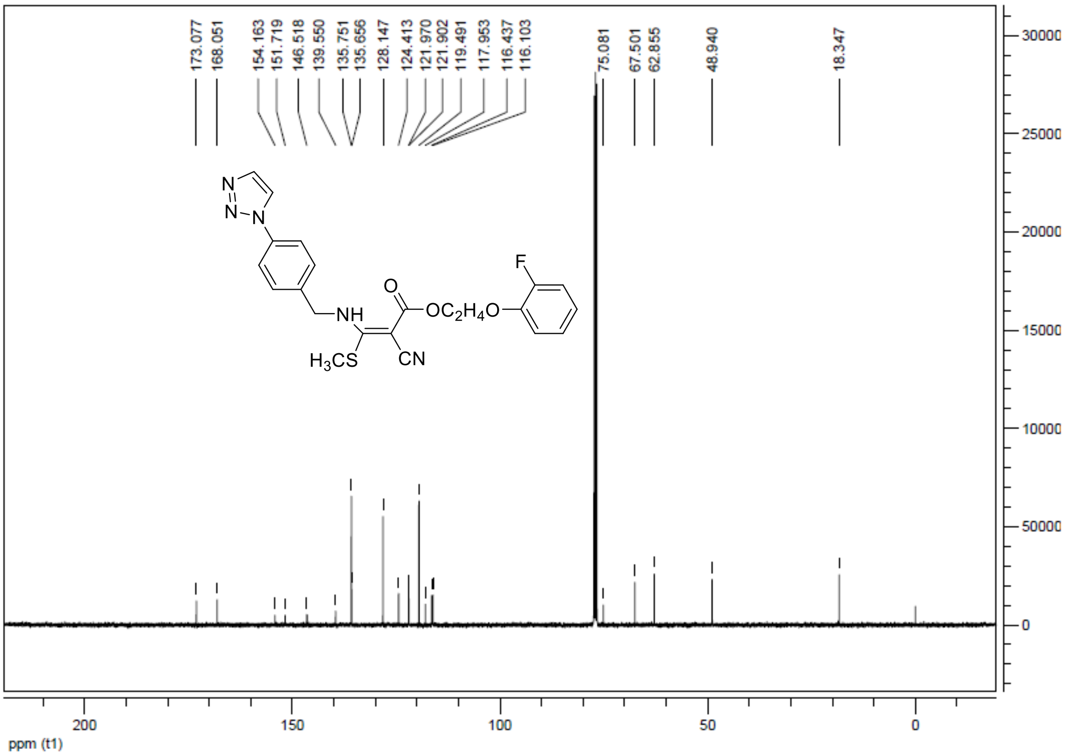

Figure S24. ^{13}C-NMR of compound **9h** (100 MHz, $CDCl_3$)

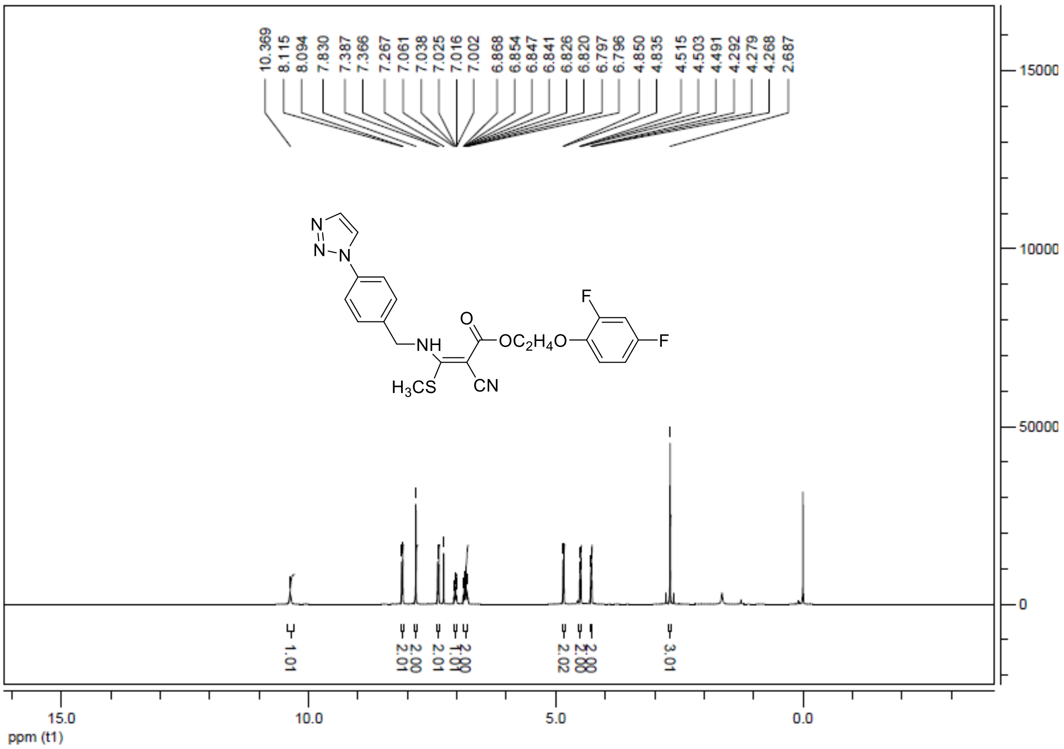

Figure S25. ^{1}H-NMR of compound **9i** (400 MHz, $CDCl_3$)

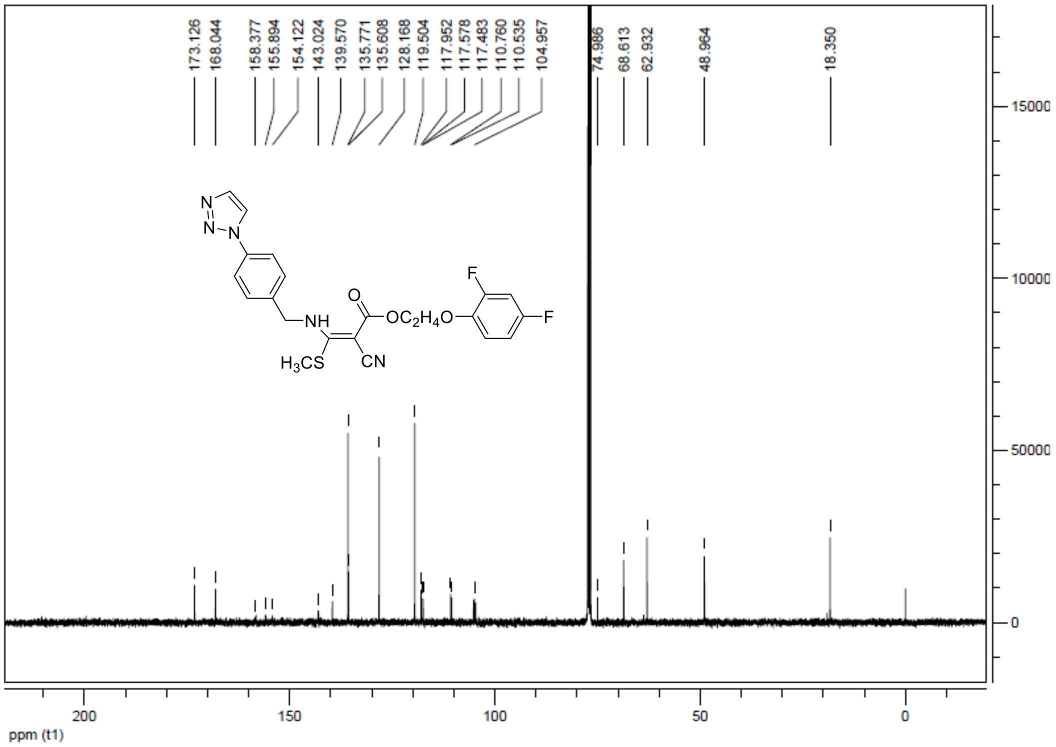

Figure S26. ^{13}C-NMR of compound **9i** (100 MHz, $CDCl_3$)

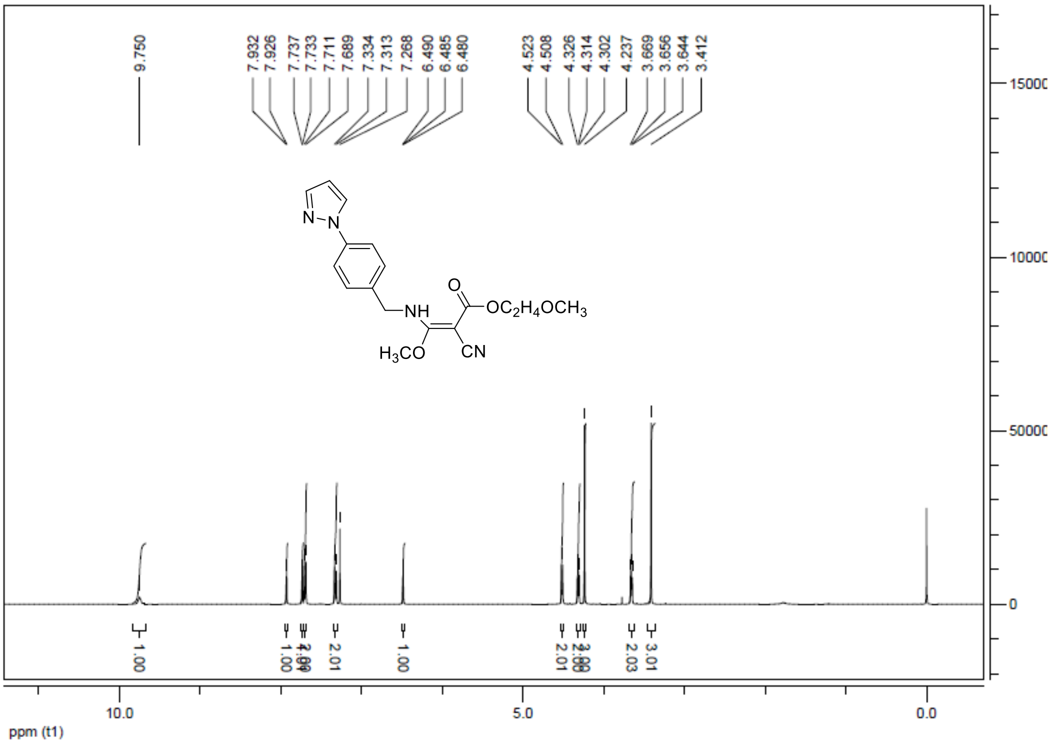

Figure S27. ^{1}H-NMR of compound **10a** (400 MHz, $CDCl_3$)

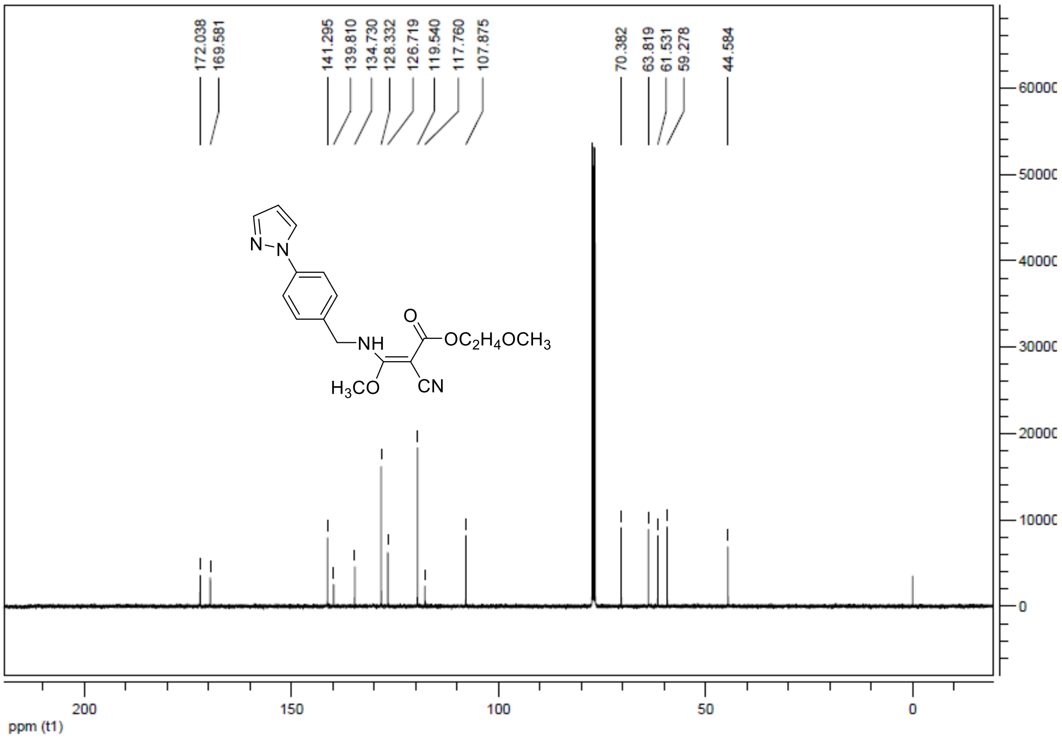

Figure S28. ^{13}C-NMR of compound **10a** (100 MHz, $CDCl_3$)

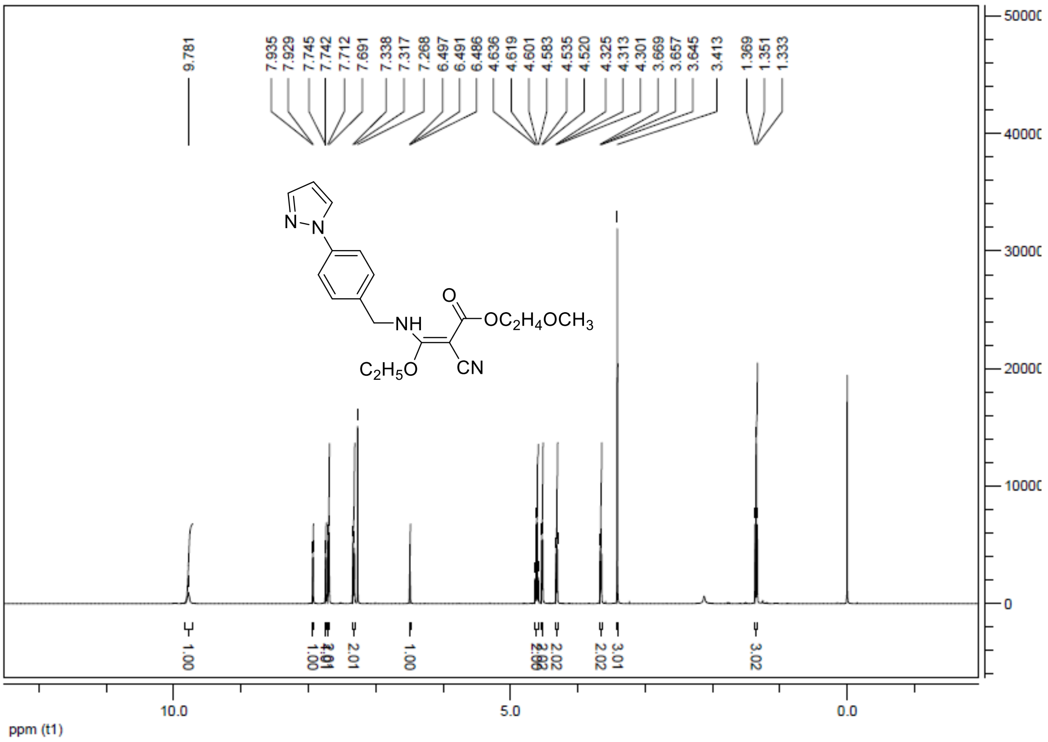

Figure S29. ^{1}H-NMR of compound **10b** (400 MHz, $CDCl_3$)

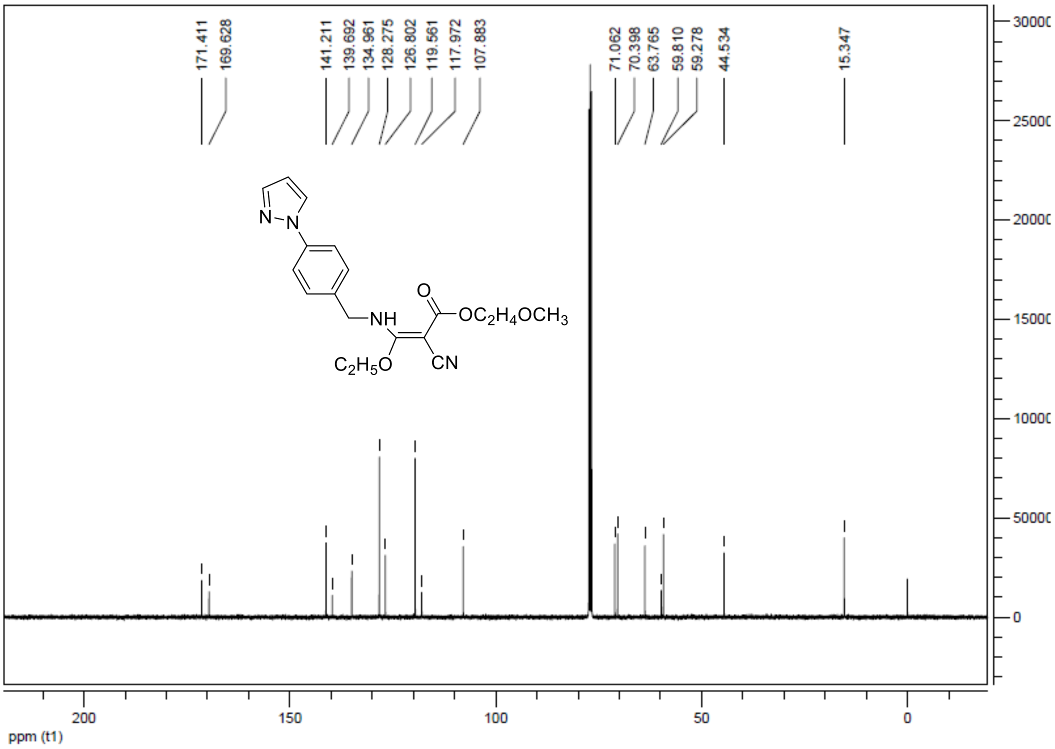

Figure S30. ^{13}C-NMR of compound **10b** (100 MHz, $CDCl_3$)

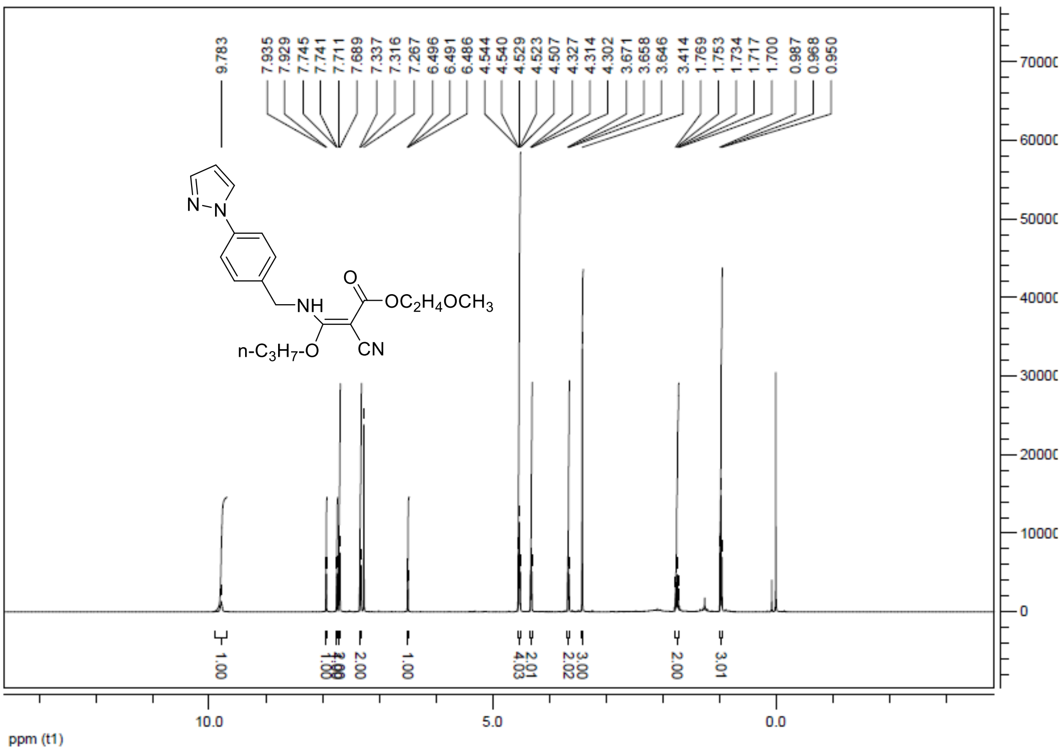

Figure S31. ^{1}H-NMR of compound **10c** (400 MHz, $CDCl_3$)

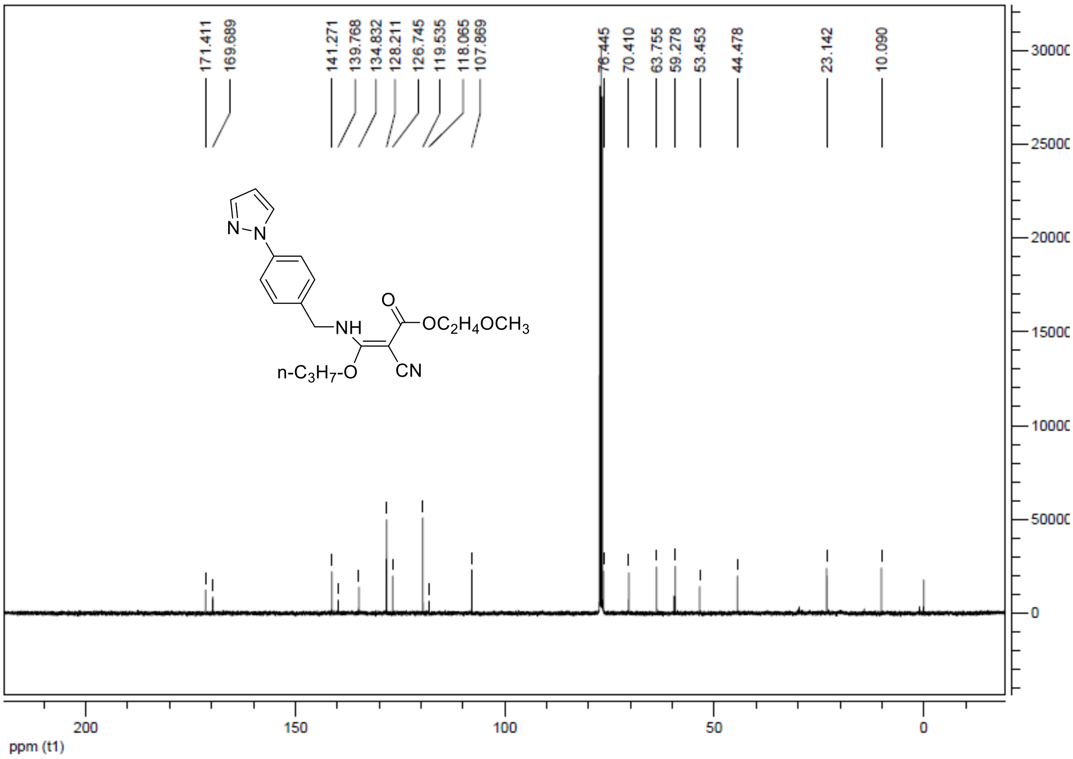

Figure S32. ^{13}C-NMR of compound **10c** (100 MHz, $CDCl_3$)

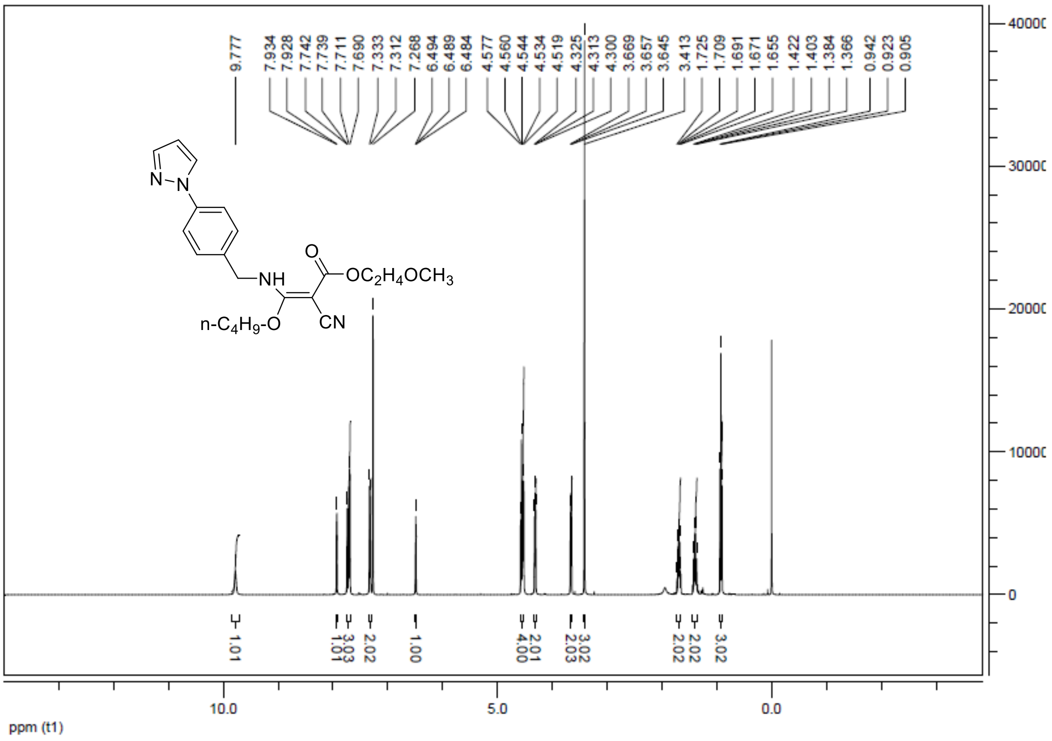

Figure S33. ^{1}H-NMR of compound **10d** (400 MHz, $CDCl_3$)

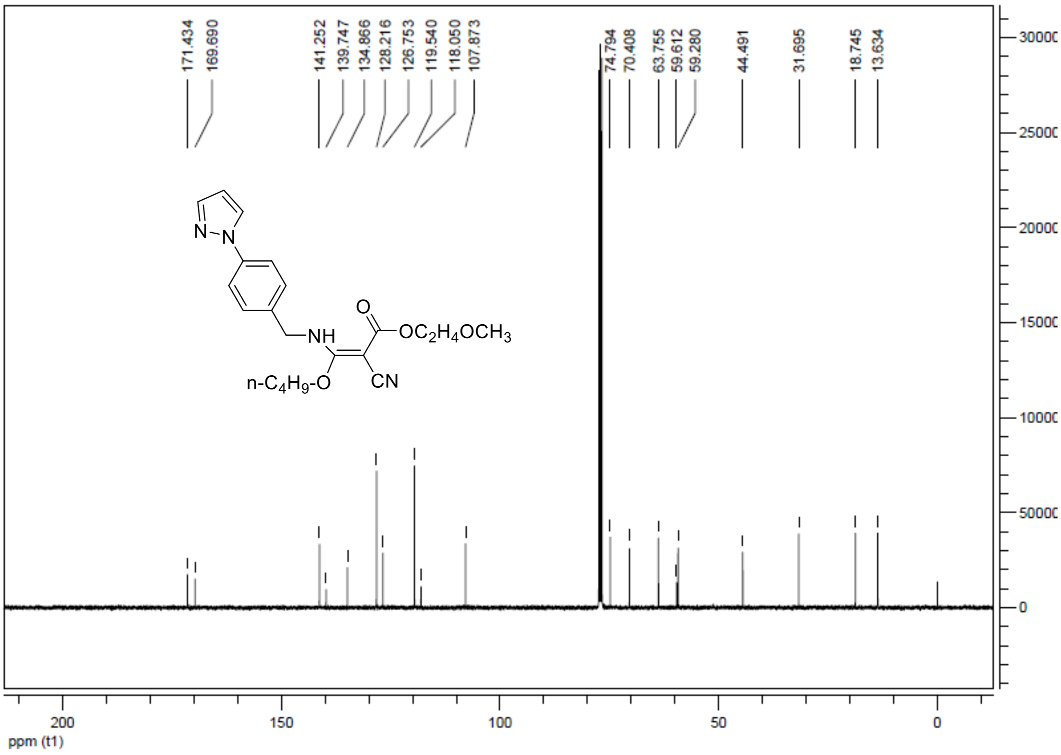

Figure S34. ^{13}C-NMR of compound **10d** (100 MHz, $CDCl_3$)

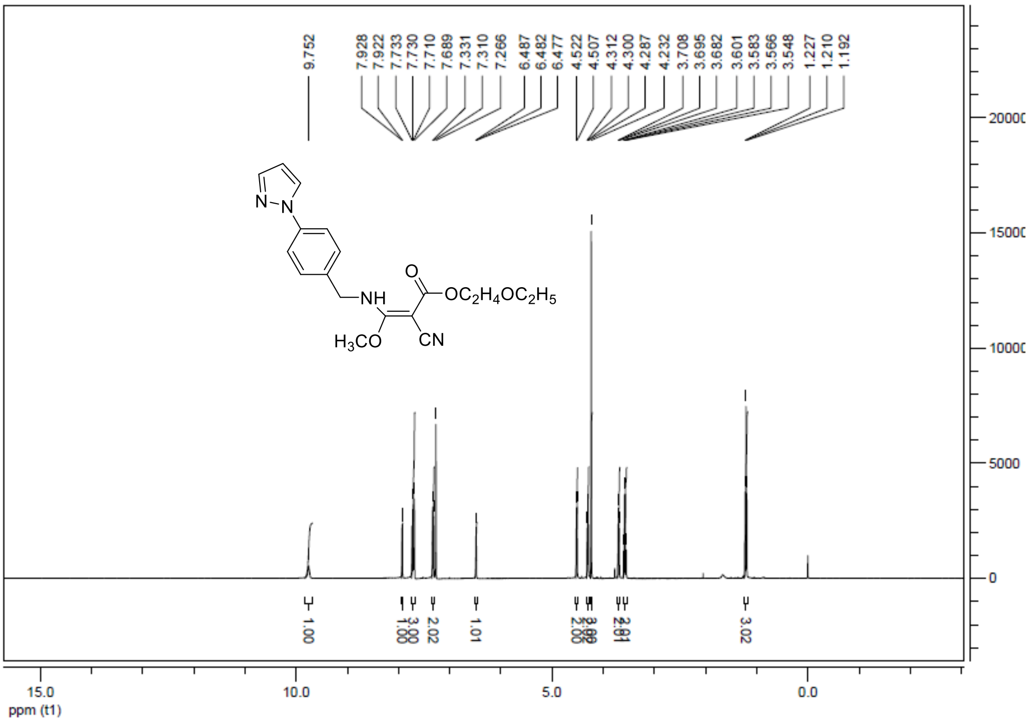

Figure S35. ^{1}H-NMR of compound **10e** (400 MHz, $CDCl_3$)

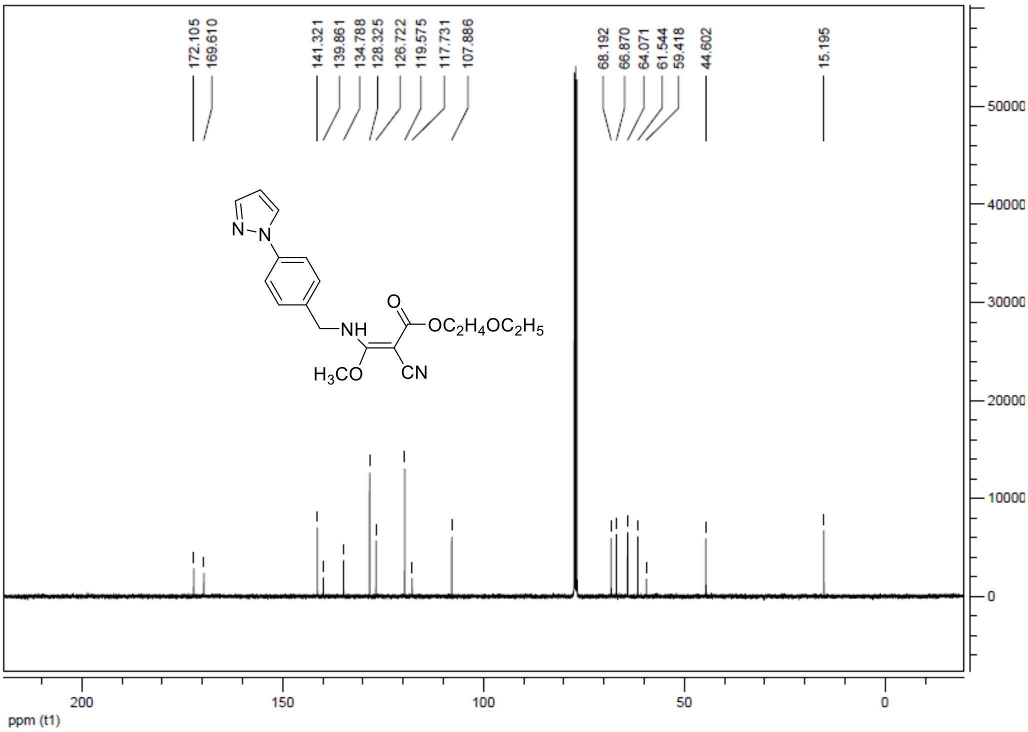

Figure S36. ^{13}C-NMR of compound **10e** (100 MHz, $CDCl_3$)

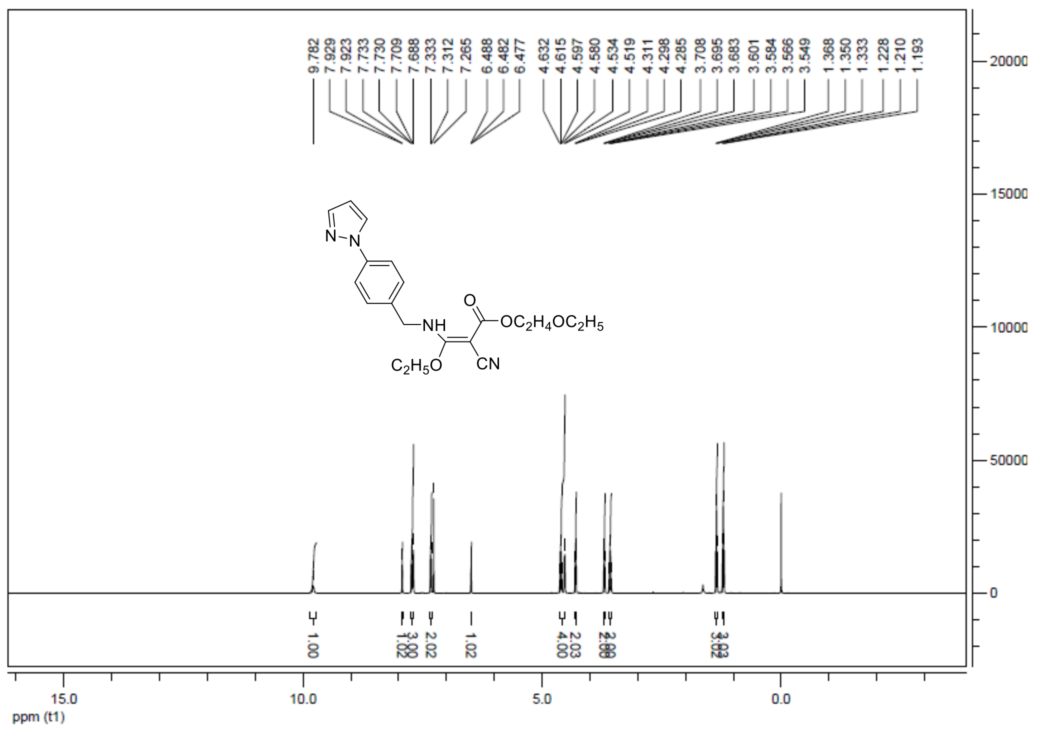

Figure S37. ^{1}H-NMR of compound **10f** (400 MHz, $CDCl_3$)

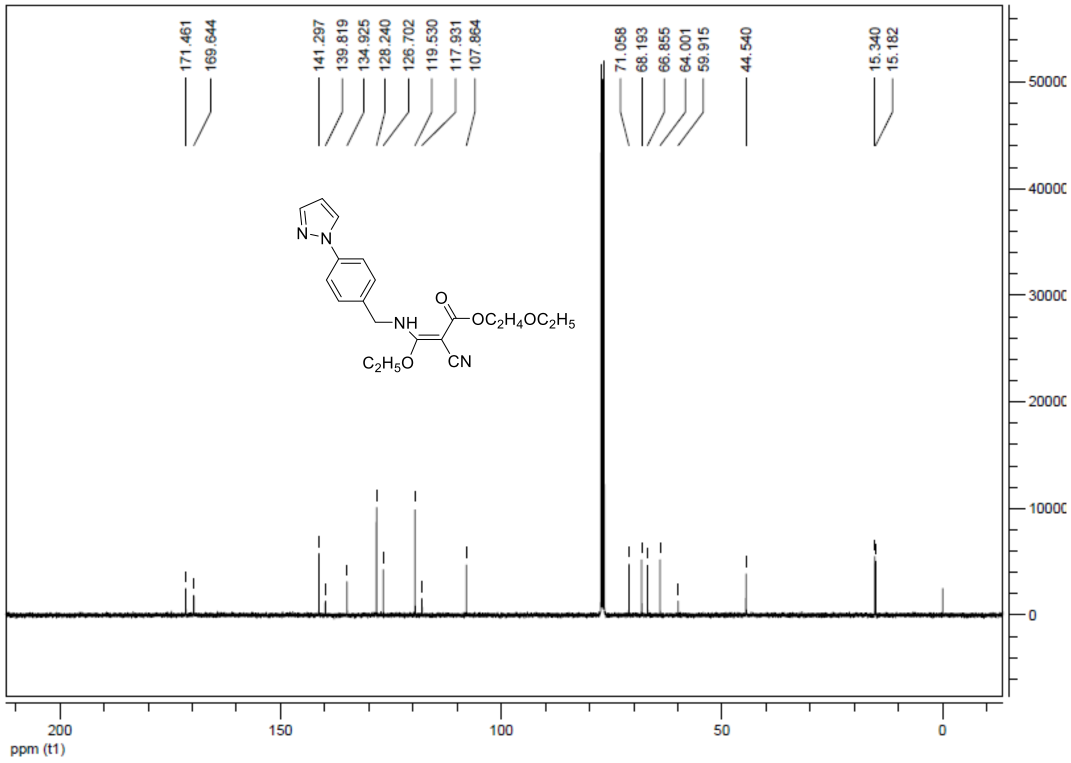

Figure S38. ^{13}C-NMR of compound **10f** (100 MHz, $CDCl_3$)

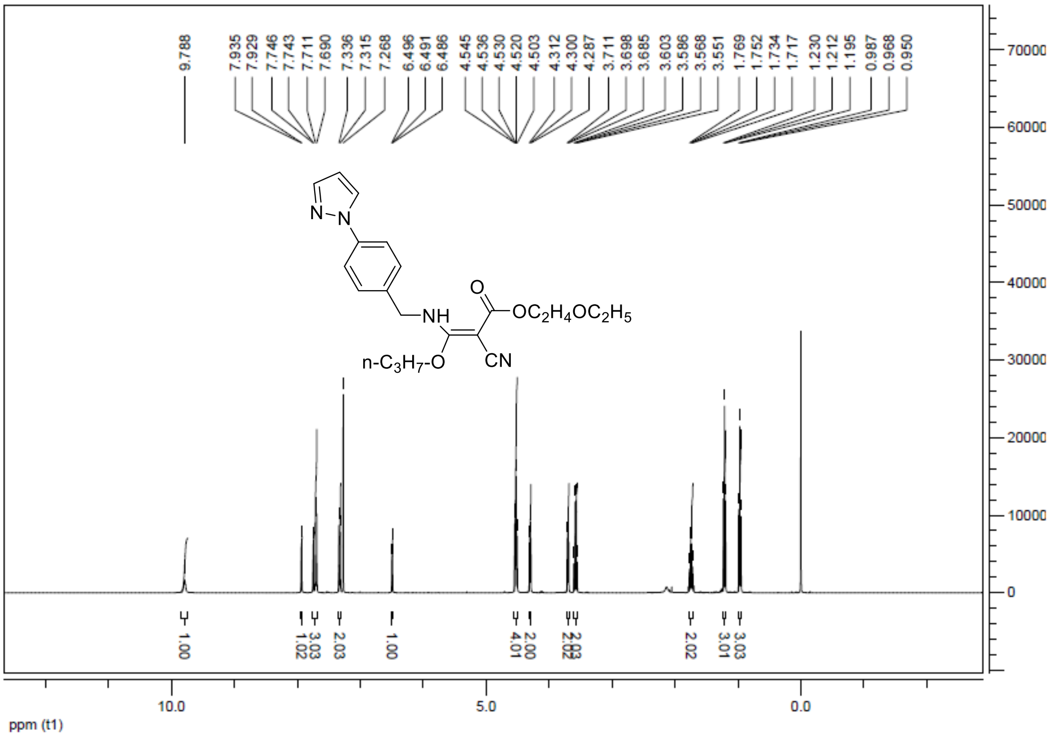

Figure S39. ^{1}H-NMR of compound **10g** (400 MHz, $CDCl_3$)

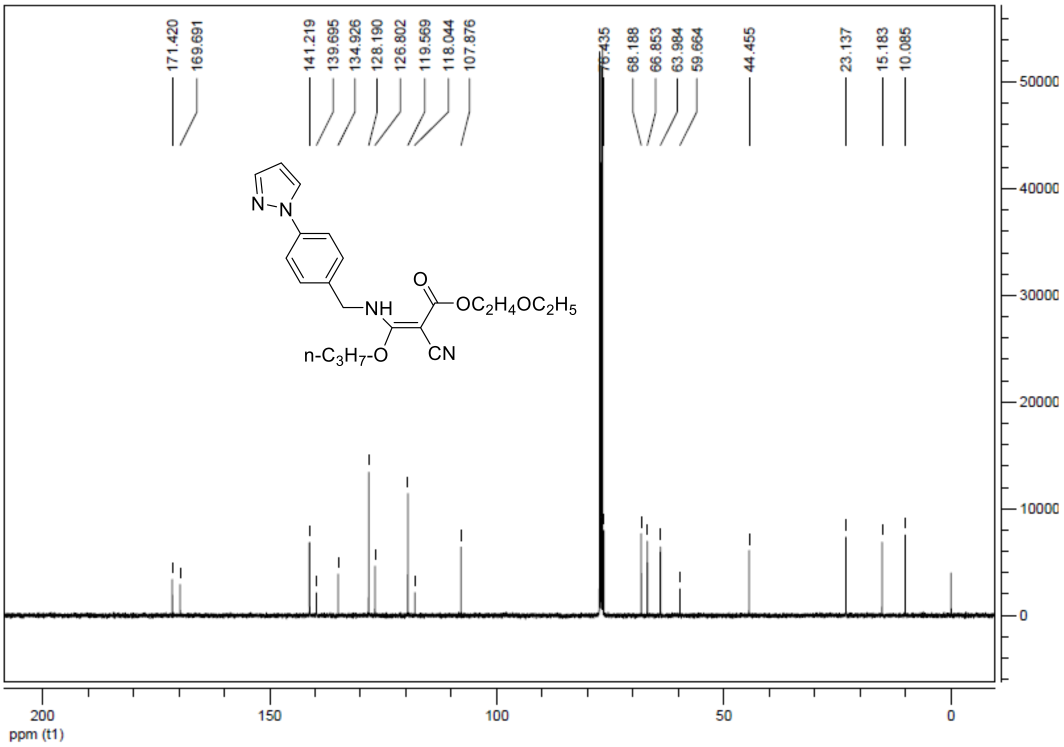

Figure S40. ^{13}C-NMR of compound **10g** (100 MHz, $CDCl_3$)

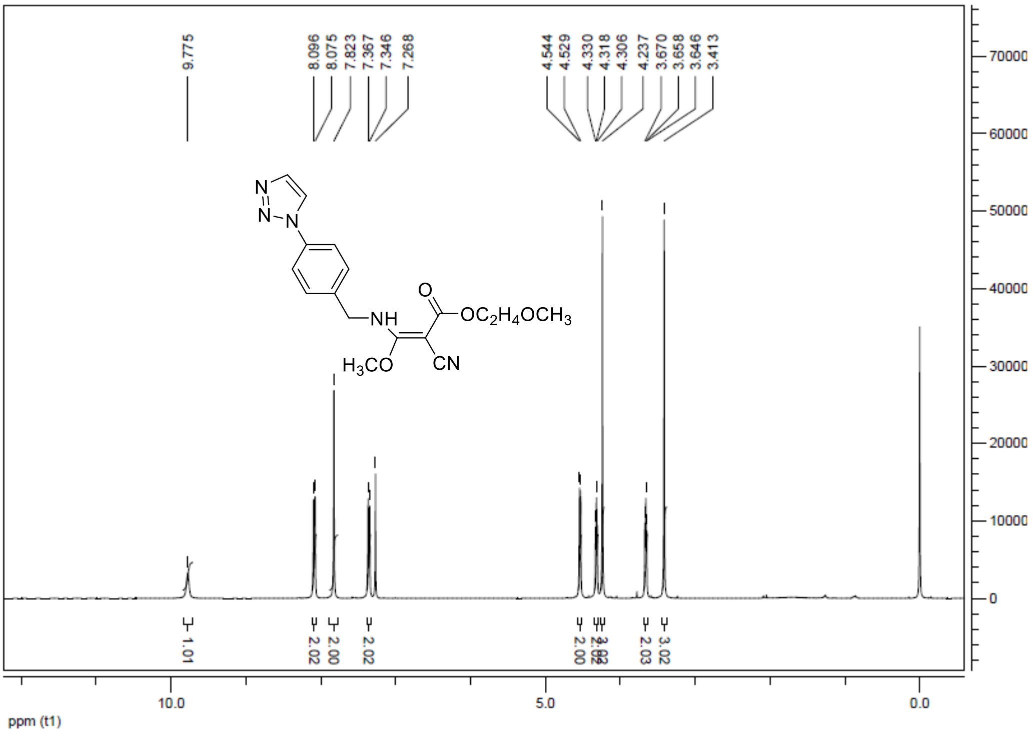

Figure S41. ^{1}H-NMR of compound **10h** (400 MHz, $CDCl_3$)

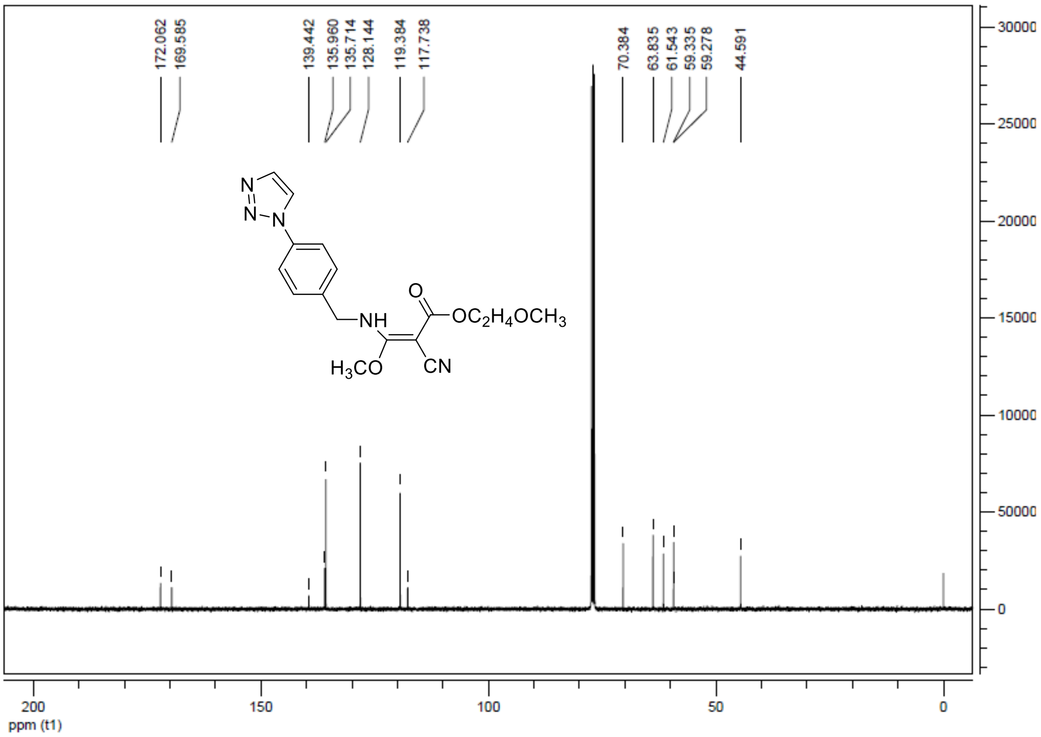

Figure S42. ^{13}C-NMR of compound **10h** (100 MHz, $CDCl_3$)

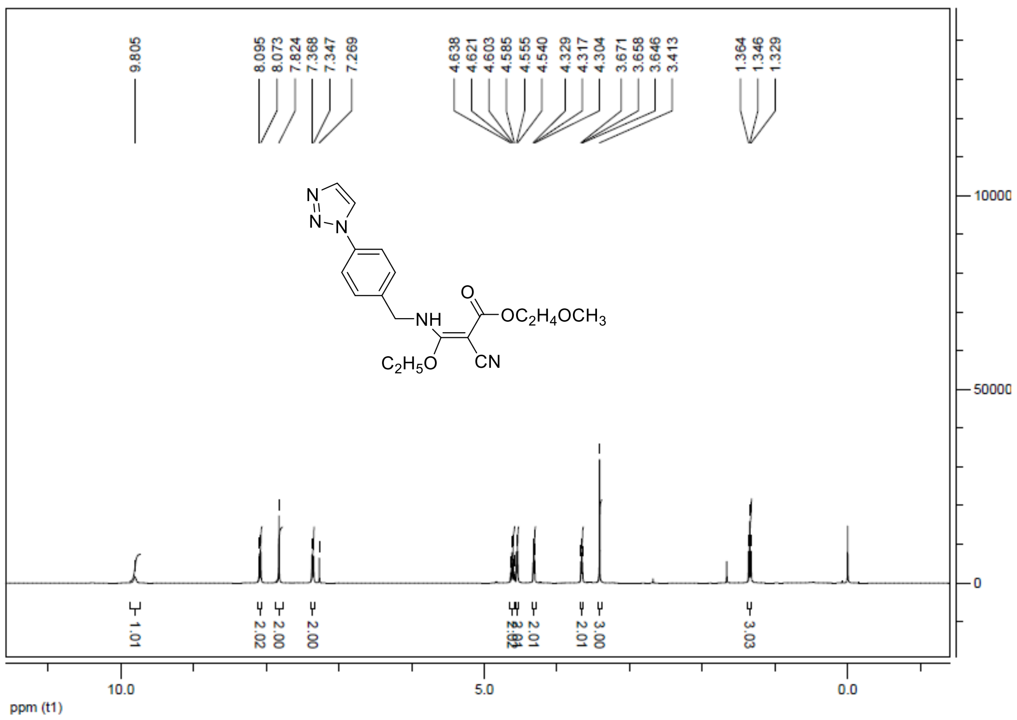

Figure S43. ^{1}H-NMR of compound **10i** (400 MHz, $CDCl_3$)

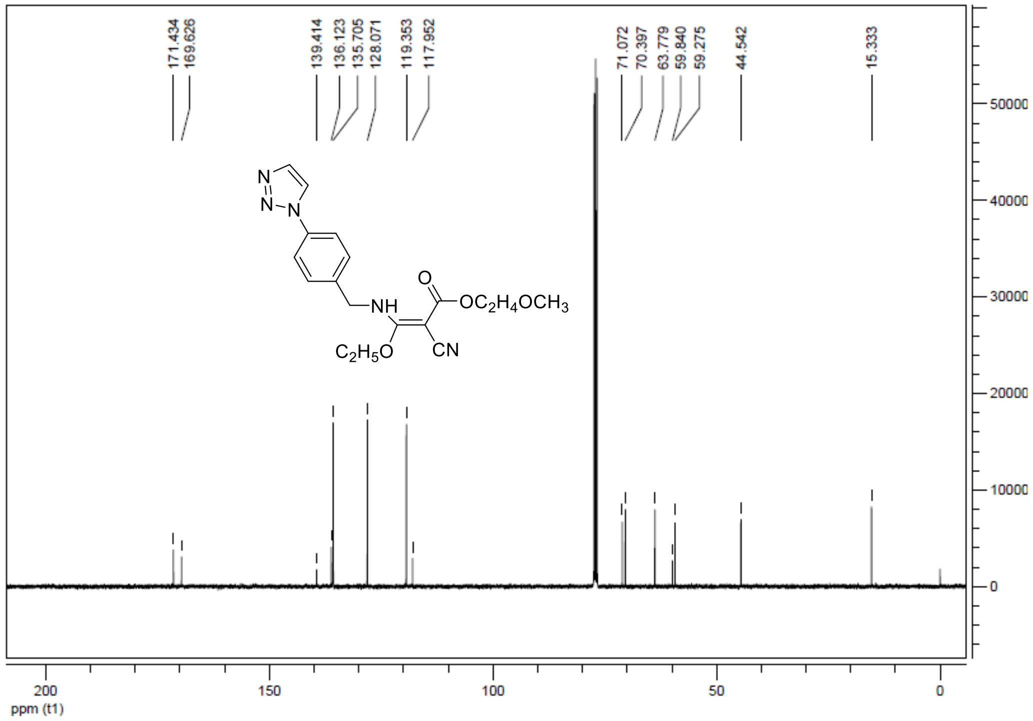

Figure S44. ^{13}C-NMR of compound **10i** (100 MHz, $CDCl_3$)

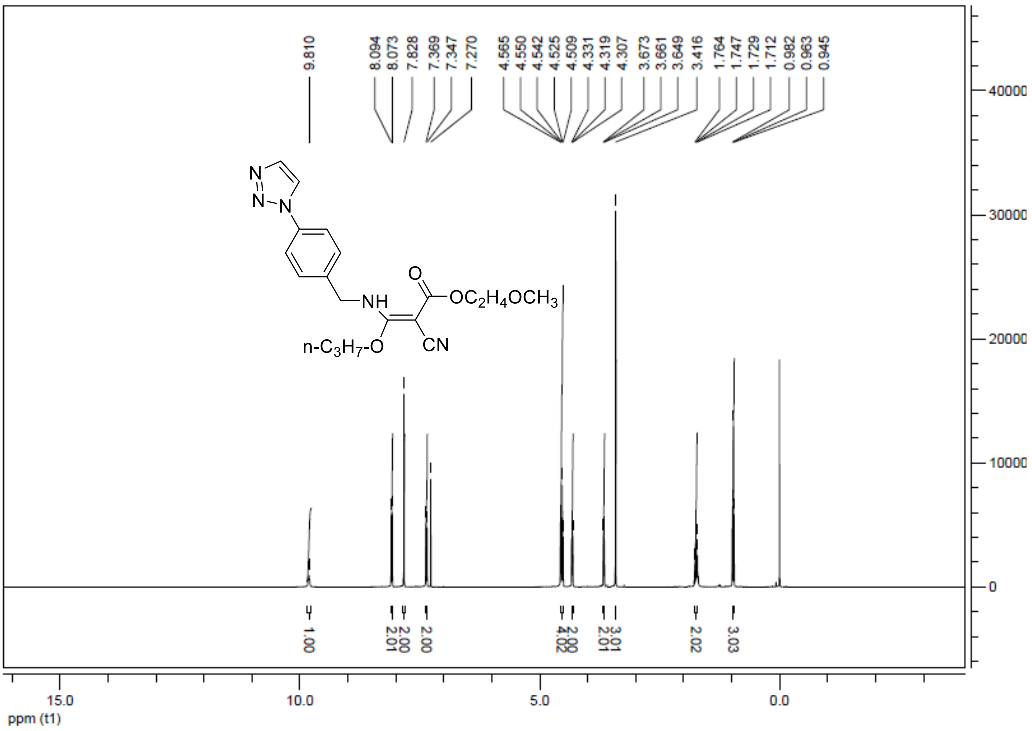

Figure S45. ^{1}H-NMR of compound **10j** (400 MHz, $CDCl_3$)

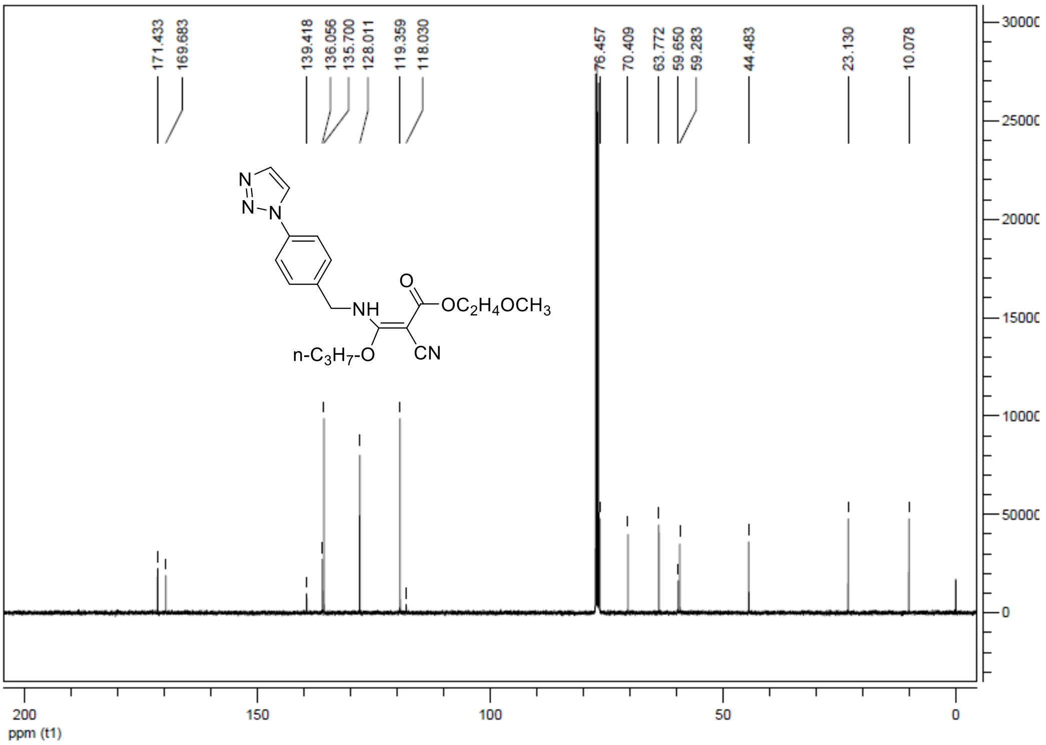

Figure S46. ^{13}C-NMR of compound **10j** (100 MHz, $CDCl_3$)

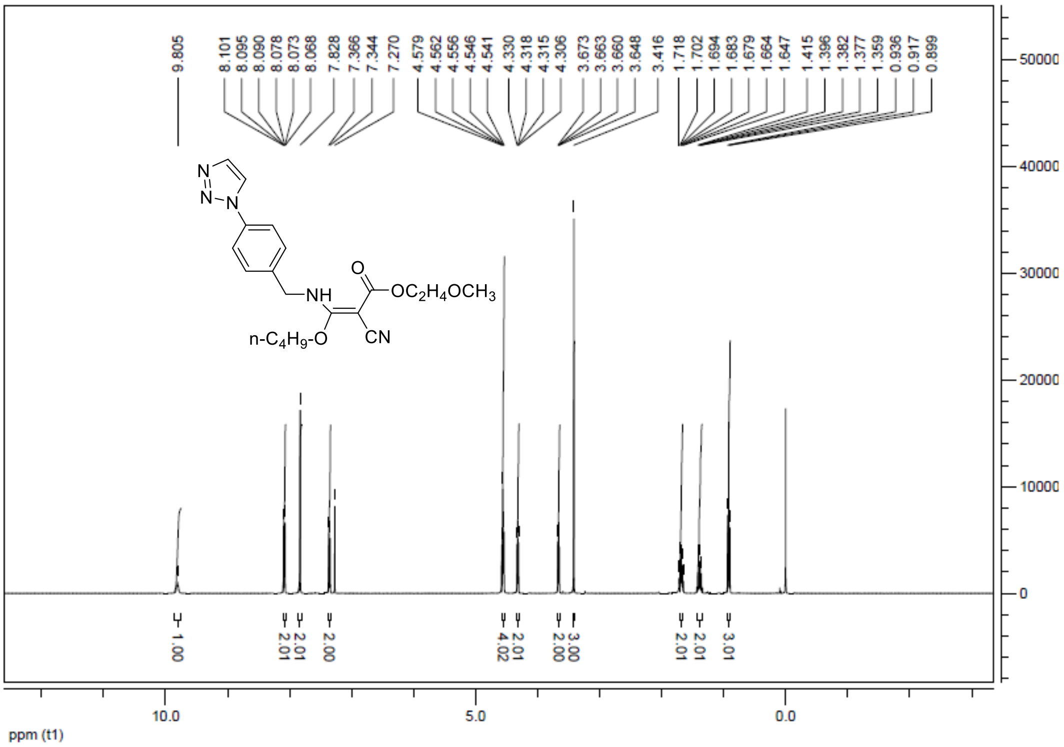

Figure S47. ^{1}H-NMR of compound **10k** (400 MHz, $CDCl_3$)

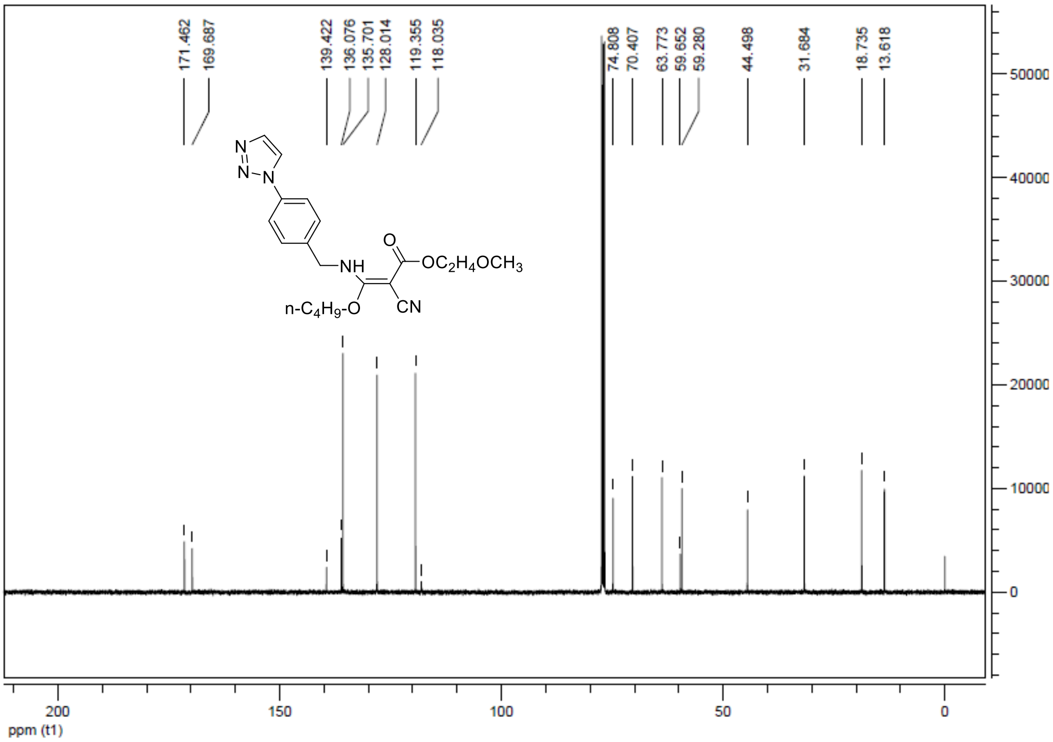

Figure S48. ^{13}C-NMR of compound **10k** (100 MHz, $CDCl_3$)

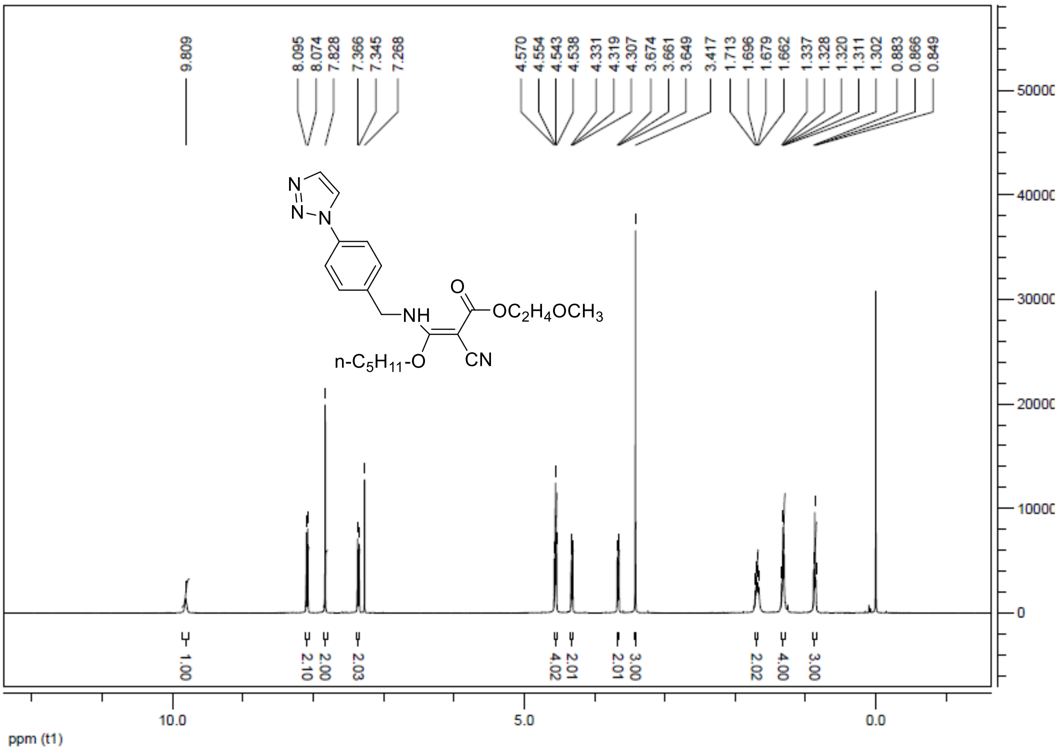

Figure S49. ^{1}H-NMR of compound **10l** (400 MHz, $CDCl_3$)

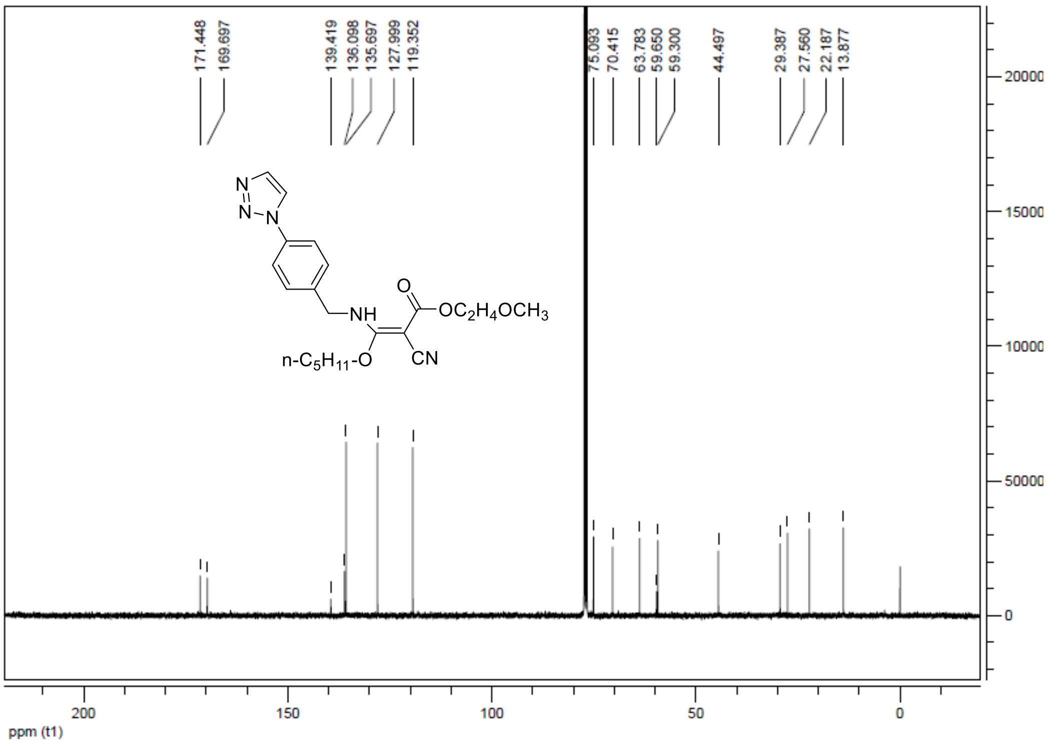

Figure S50. ^{13}C-NMR of compound **10l** (100 MHz, $CDCl_3$)

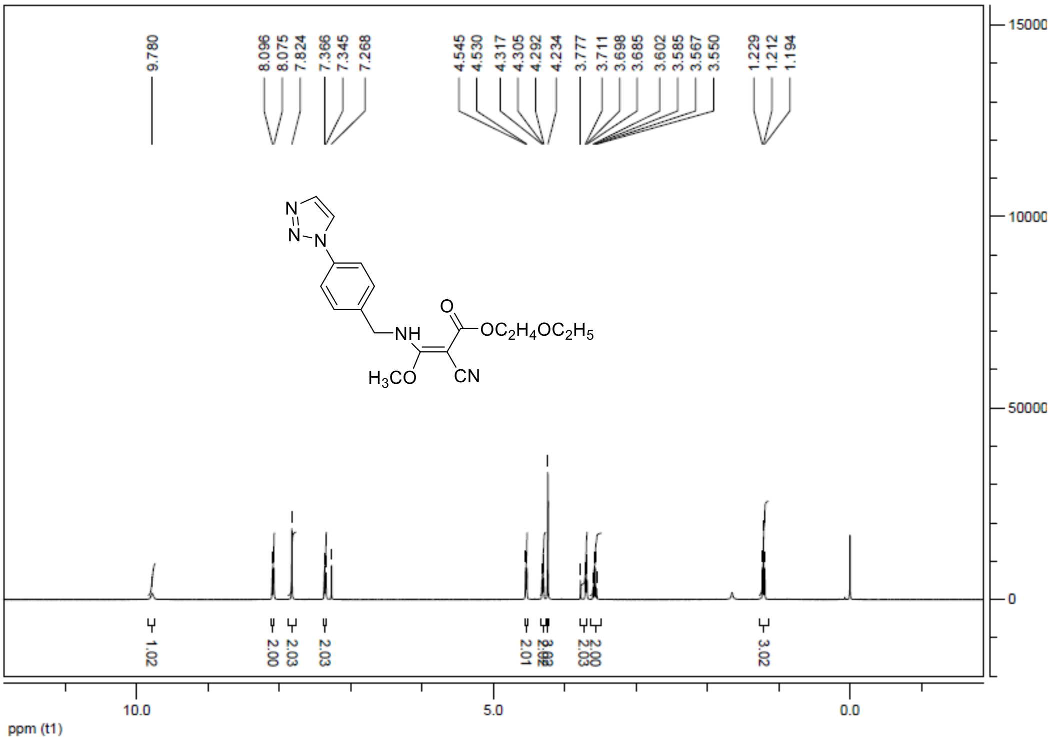

Figure S51. ^{1}H-NMR of compound **10m** (400 MHz, $CDCl_3$)

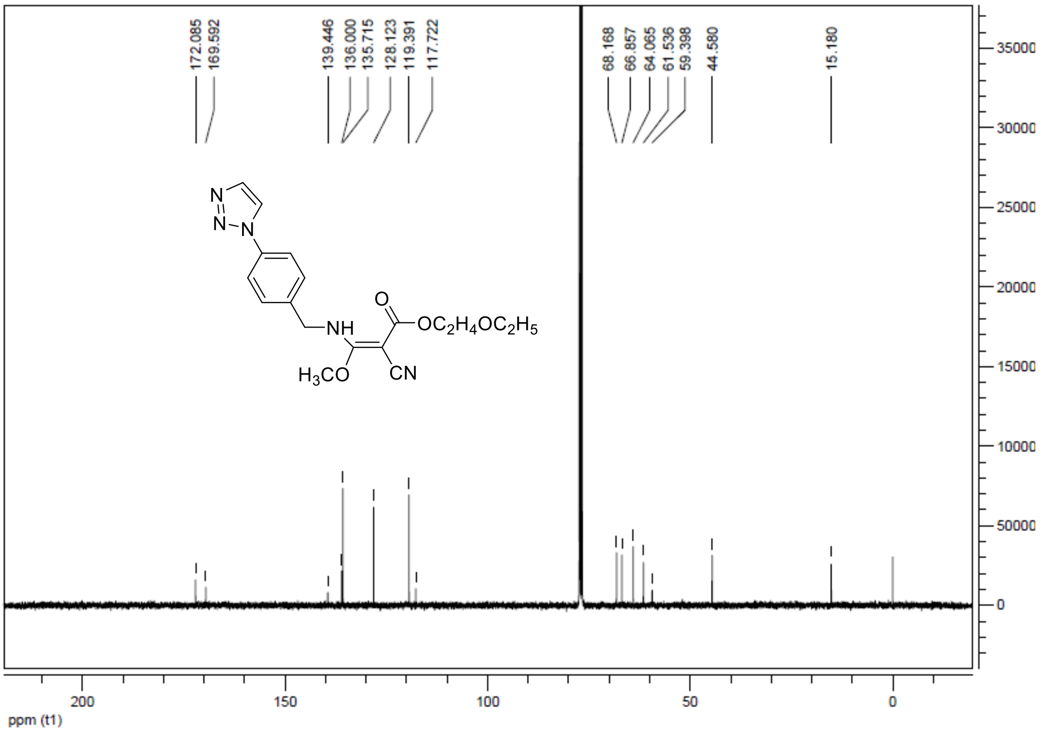

Figure S52. ^{13}C-NMR of compound **10m** (100 MHz, $CDCl_3$)

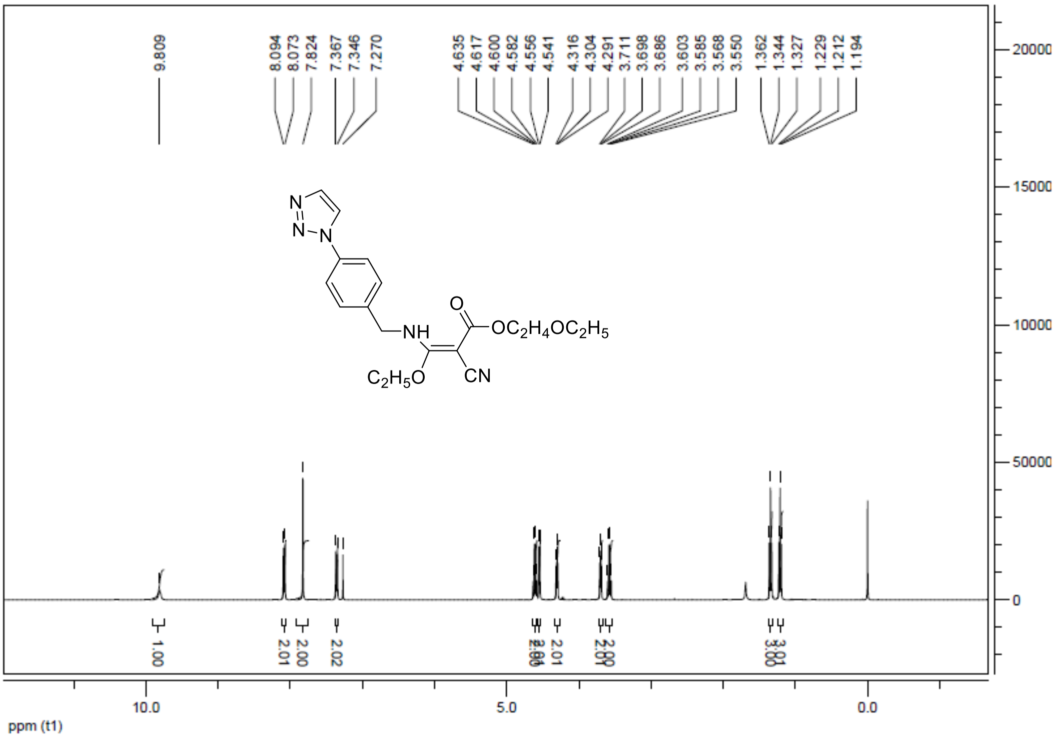

Figure S53. ^{1}H-NMR of compound **10n** (400 MHz, $CDCl_3$)

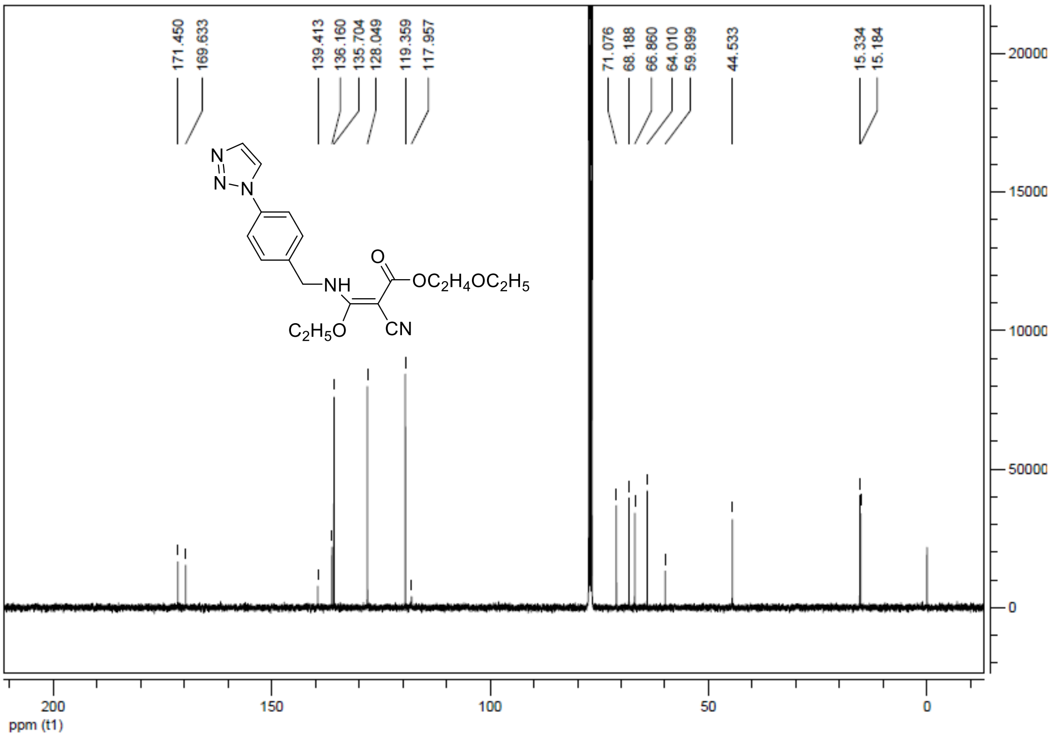

Figure S54. ^{13}C-NMR of compound **10n** (100 MHz, $CDCl_3$)

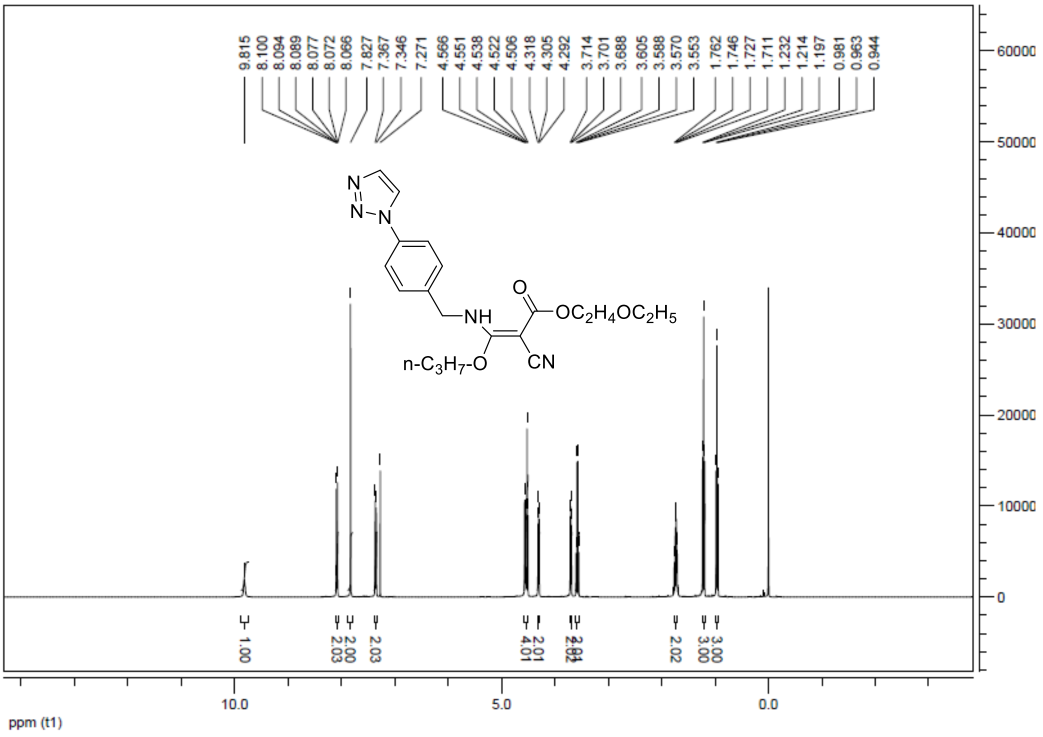

Figure S55. ^{1}H-NMR of compound **10o** (400 MHz, $CDCl_3$)

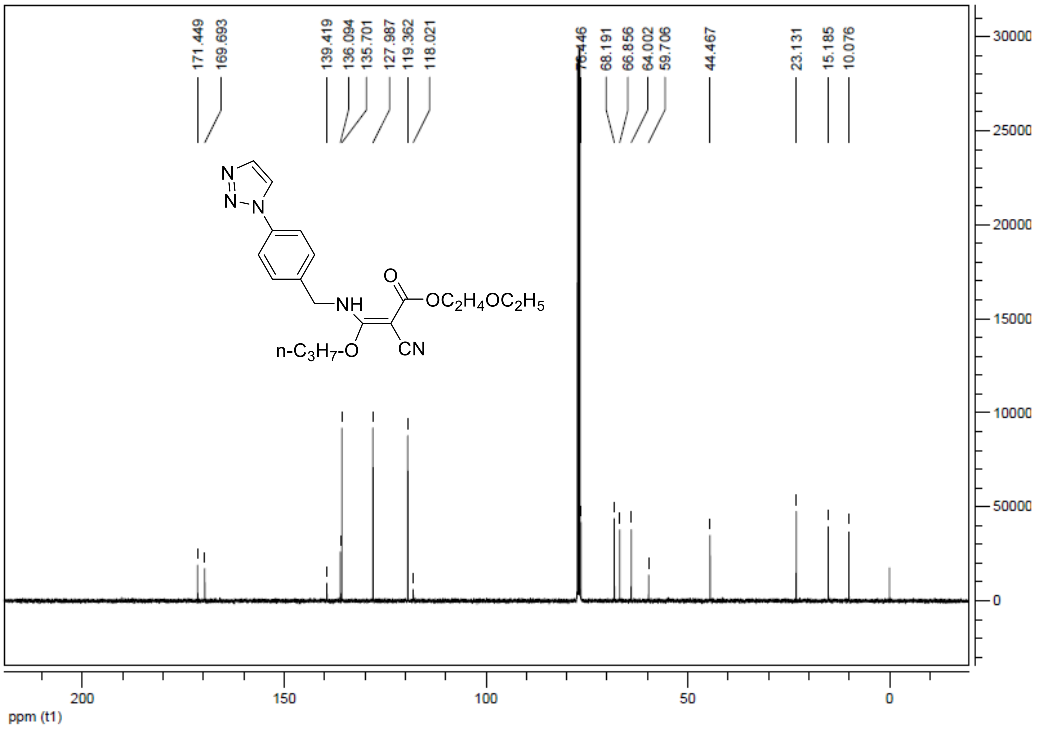

Figure S56. ^{13}C-NMR of compound **10o** (100 MHz, $CDCl_3$)
